# Effect of temperature on hydrothermal liquefaction of high lipids and carbohydrates content municipal primary sludge

**DOI:** 10.1016/j.heliyon.2024.e24731

**Published:** 2024-01-19

**Authors:** Jacky Cheikhwafa, Katarzyna Glińska, Esther Torrens, Christophe Bengoa

**Affiliations:** Universitat Rovira i Virgili, Departament d’Enginyeria Química, Avinguda dels Països Catalans 26, 43007, Tarragona, Spain

**Keywords:** Municipal primary sludge (PS), Hydrothermal liquefaction (HTL), Biocrude separation into oils and asphaltenes, Full characterization of all phases

## Abstract

The study assessed the valorisation of primary sludge through HTL and the influence of temperature on the product distribution. The experiments were conducted at different temperatures, 30 min reaction time, and 100 rpm stirring rate. The maximum yield of biocrude produced was 39.47% at 270 °C. The best yield of oils was 23.96% at 300 °C. The lowest yield of asphaltenes was 12.50% at 240 °C. HHV for biocrude were always between 39 and 41 MJ/kg, close to petroleum. Best energy recovery for biocrude was 82% at 270 °C.

## 1 Introduction

In the countries of the European community, the production of sludge will exceed largely 13.0 million tons predicted for 2020 [1]. The common ways for sludge disposal are the: landfilling, land disposal, land application and incineration [2]. Nevertheless, the environmental problems created by these routes, promoted the necessity to well manage the disposal of sewage sludge, and look for alternatives as its reuse or valorisation [1]. The recycle or valuation of sewage sludge has become a domain of advancing research and investment around the world [3].

Hydrothermal liquefaction is one of the most widely used sustainable transformation processes of sewage sludge is [4]. Hydrothermal liquefaction (HTL) is a thermochemical depolymerization approach that converts organics contained in wet biomass, - moisture content of sludge is between 95 and 98% [5] - into high-valuable products [6]. The high amount of water contained in sewage sludge plays one of the principal roles, because it is the reaction medium and, catalyses the thermochemical process. For these reasons, hydrothermal liquefaction, is a suitable option for handling high moisture content solids, where water is used as the medium for breaking down the organic matter into simpler chemicals at high temperatures and pressures [7]. Usually, hydrothermal liquefaction operates at a temperature ranging from 280 to 400 °C and under pressure from 10 to 25 MPa [8]. At these conditions, water is near its critical point, and acts as a non-polar solvent, being an extremely effective reaction medium for the transformation of organics. Sewage sludge is principally converted into biocrude (main product) and three by-products, namely, biochar, aqueous phase, and biogas [2]. Hydrothermal liquefaction has proven that it is a valorising process able to produce energetically dense biocrude oils from wet feedstocks without any previous removal of water [9]. Additionally, as the drying step of the sludge can be skipped, this contributes to important cost savings [5].

For all these reasons, sewage sludge can be considered as a valuable feedstock because its continuous availability, its interesting composition (lipids, proteins, carbohydrates and lignin), or its high calorific value [8,10]. Moreover, hydrothermal liquefaction, is able to recover a larger part of the chemical energy contained by the molecules that constitute the sewage sludge [11].

Primary sludge is a thick fluid, grey in colour, slimy and with highly ghastly odours. Primary sludge is drawn at solids concentrations of 2–6 % out of which 55–70 % are organic as lipids, proteins and carbohydrates [3]. In fact, primary sludge has more than 40% of proteins and carbohydrates [12]. Specifically, the primary sludge from the Reus treatment plant (Spain) has had a fairly constant composition over the last few years: 20–30 % ashes; 20–30 % lipids; 20–30 % protein; 20–30 % carbohydrates [13,14]. During the HTL process, the biomass of the primary sludge, rich in carbohydrates, lipids and proteins, in compressed and hot water, reacts very quickly and efficiently through depolymerization, isomerization, reforming and repolymerization reactions (Bischt et al., 2022).

Biocrude and by-products yields are distributed differently depending on the temperature. The reaction temperature is one of the parameters that have a significant influence on HTL products distribution [8]. The yield of biocrude increases progressively with the increase of temperature until reaching a maximum value. When this value is exceeded, the excessive temperature provokes the cracking of the biocrude phase into gaseous products and the formation of high molecules by additional re-polymerization [15]. In the HTL process, the increase in temperature implies an increase in efficiency. This is maximum in a relatively low temperature range, between 250 and 350 °C, and high biocrude yields are obtained [16]. But, if at low temperature you work with a short reaction time, this promotes the formation of heavy biocrude [17].

When the temperature approaches or exceeds the critical temperature of the water, two processes arise that limit the liquefaction and reduce the yield of biocrude. First, excessive reaction temperature favours repolymerization and decomposition of reaction intermediates. Secondary reactions then appear that transform the intermediate products accelerating the generation of additional hydrocarbon gases. Secondly, the high temperatures close to the critical temperature of the water drastically change the characteristics of the water, accumulating large amounts of free radicals that stimulates the reactions of polymerization, condensation and cyclization. These are responsible for the increase in the yield of gases and biochar and the decrease in the yield of biocrude (Bischt et al., 2022).

The biocrude is deep dark brown, close to black, and has a high viscosity like bitumen or distillation vacuum residue. On the other hand, biocrude has in its composition a high content of heteroatoms, like oxygen or nitrogen. This fact is common in biocrudes from almost all types of biomasses treated by hydrothermal liquefaction [18].

To our knowledge, to date, few studies have focused on the study of HTL of primary sludge. The most recent one makes a comparison between all types of sludge from four Danish treatment plants [19]. The results with primary sludge have ranged between 28.2 and 46.8% on a dry basis, or what is the same based on total solids. The authors declared that the four treatment plants have primary sludge with very different compositions and this, has conditioned the biocrude yield. In an older work, where the effect of catalysts on the HTL of primary sludge is compared, a yield of 30.7% based on volatile solids is reached, in the case of not using a catalyst [20]. Previously, in a continuous HTL process, a biocrude yield of 37.3% based on volatile solids has been reached [21]. These are the recent studies carried out on the effect of temperature on the HTL of primary sludge from urban treatment plants. It is evident that it is still necessary to study the valorisation of primary sludge in energy vectors or other applications.

The excessive production of municipal sludge along with the environmental problems generated from its disposal urge its valorisation. Even though many factors can affect the products of HTL, it is known that temperature plays the key role in biocrude production. At high temperature, lipids, carbohydrates, proteins, lignin, cellulose and hemicellulose are degraded and transformed to biocrude [2]. However, none has studied the HTL of primary sludge, containing very low solid content (around 5%), as received, without any addition of water. Therefore, the objective of this work is to convert primary sludge, containing high lipid and carbohydrate content, into the highest possible yield biocrude with an ultimate quality. To attain this goal, different temperature conditions were chosen and studied. In each scenario, a complete characterization of the products will be presented to establish a rigorous mass balance.

The objectives of the research work are several, firstly to verify that the primary sludge from a Mediterranean WWTP can be used for the production of biocrude. Then to verify that the characteristics of the biocrude are suitable for its direct use or needs post-treatment processes. Furthermore, the complete characterization of the other interesting phases such as biogas, rarely analysed, the aqueous phase and biochar, should allow their post-valuation as, for example, energy vectors or processes for treating contaminated soils.

The influence of temperature on biocrude yield and product distribution will be studied, in order to maximise yield and quality. Finally, a complete characterization of the products will be presented to establish a rigorous mass balance.

## 2 Materials and methods

### 2.1 Reagents

Dichloromethane 99.9 % (ref.: 32,222), Toluene 99.7 % (ref.: 32,249) and 2-Propanol 99.9 % (ref.: 59,300) were purchased from Honeywell, Germany. Methanol (ref.: 412,722), HPLC-GOLD-Ultragradient grade, was brought from Carlo Erba reagents, Italy. n-Hexane 95 % (ref.: 363,242), high performance chromatography grade, and phenol crystalline (ref: 144852.1211) and n-heptane (ref.: 162062.1611) were provided by PanReacAppliChem, Spain. Sulfuric acid reagent (ref: 34,632), orange reagent (ref: 131130.1612), sulfuric acid 95.0–97.0 % (ref: 30,743), bovine serum albumin (BSA) (ref: A9647), sodium hydroxide 98 % (ref: 30,620), sodium carbonate (ref: 222,321), potassium sodium tartrate tetrahydrate (ref: 217,255), copper (II) sulphate pentahydrate (ref: 209,198), Folin&Ciocalteu's phenol reagent (ref: F9252), magnesium sulphate monohydrate (ref: 434,183), anhydrous sodium sulphate (ref: 239,313) and fuming hydrochloric acid (ref: 84,418), high analytical reagent grade, were supplied by Sigma – Aldrich, USA. ASTM® D2887 Reference Gas Oil (ref: 48,873) and chloroform-d (ref: 225,789) provided also by Sigma Aldrich, USA.

### 2.2 Primary sludge collection and managing

Samples of primary sludge were provided by the municipal wastewater treatment plant of Reus in Tarragona, Spain. The capacity of WWTP reaches 25,000 m^3^/day of wastewater to treat. 500 mL bottles of primary sludge were sampled after partial gravity thickening, located after the primary decanter. They were stored in a freezer at −15 °C and defrosted in an oven at 60 °C for 5 h. The bottles of primary sludge were used directly as received.

### 2.3 Characterization of primary sludge

**Fig. 1:**
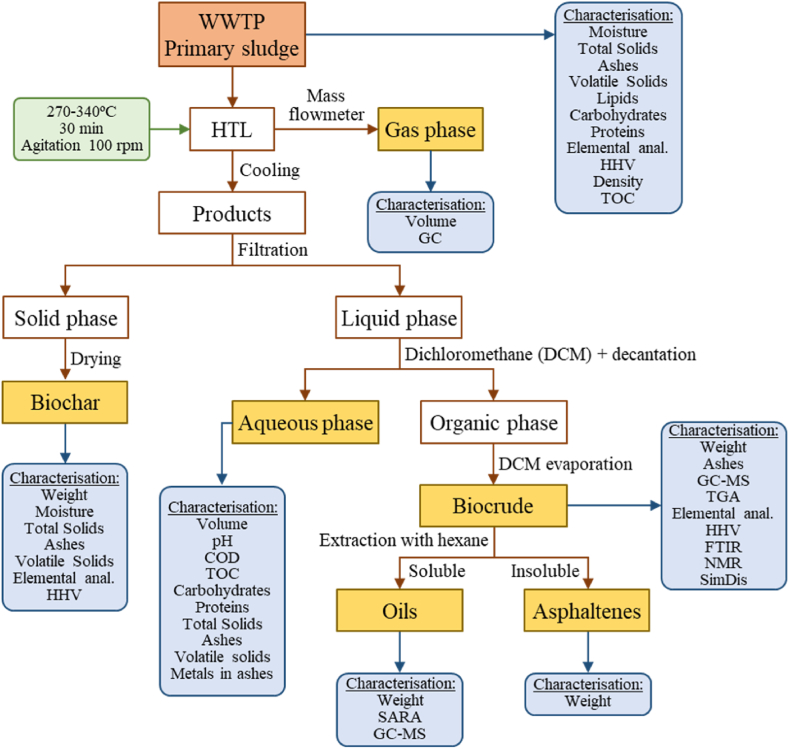
Separation methodology of products of HTL of primary sludge.

Fig. 1 shows the analytical techniques performed to fully characterise the primary sludge. The characterisation of primary sludge was carried out in triplicate.

Total solids (TS), volatile solids (VS) and ash content were measured according to standard methods 2540B and 2540E respectively [22]. The extraction of lipids was achieved in a Soxhlet apparatus using hexane as a solvent, according to standard method 5520E [22].

Total carbohydrates percentage was determined by phenol-sulfuric acid Dubois method [23]. Shortly, 0.05 mL of 80% phenol solution was added to 2 mL of diluted sludge sample in a glass tube. Then, 5 mL concentrated sulfuric acid was quickly added. The tubes were kept under room temperature for 10 min and then placed into a thermostatic bath at 30 °C another 15 min. The absorbance was measured at 480 nm.

Proteins content was determined with Lowry method [24]. The proteins solubilization in the sludge samples was carried out by heating the samples with 2 M sodium hydroxide at 100 °C for 10 min. The absorbance was measured at 750 nm.

Finally, ultimate analysis was realized by Serveis Técnics de Recerca at Universitat de Girona. Analysis was performed using an ultimate analyser (PerkinElmer model EA2400). C, H and N were determined, and O was calculated by difference. A field emission of variable pressure environmental scanning electron microscopy (ESEM) with X-ray microanalysis (Quanta 600, FEI Company), characterised by a high resolution (3 nm) was utilized to detect heavy metals of primary sludge.

### 2.4 Hydrothermal liquefaction of primary sludge

The experiments were performed in triplication in a 1 L Stainless Steel Autoclave (Autoclave Engineers model EZE Seal) with an overall size of 61.70 cm of height, encountered by a movable heating shell (maximum power: 1000 W), a fixed MagneDrive® stirrer (magnetically coupled, packless rotary impeller system) and an operating condition controller (maximum speed: 3300 rpm). The reactor is connected to a gas line through an inlet valve allowing the introduction of nitrogen. The outlet valve is linked to a gas flow meter and a Tedlar bag push lock valve 0.6 L (Superlco 30289-U) for gas collection. The rupture disc is designed to maintain 10% higher than the pressure rate. For safety and precautions, the reactor is placed in a barricade.

A bottle containing around 500 g of primary sludge weighted with a sensitive balance was emptied in the reactor. Pure nitrogen gas was purged three times to create an oxygen-free atmosphere and then pressurized up to 1 bar as an initial pressure.

In the previous literatures, temperature was selected between 200 and 450 °C and residence time between 0 and 480 min. At temperature higher than 300 °C, cracking reaction begins [2]. In terms of product yields and the energy cost, considering 300 °C as the midpoint in our study, HTL experiments were achieved at two lower conditions: 240 °C (∼33.7 bar), 270 °C (∼55.6 bar), at the midpoint 300 °C (∼86.9 bar) and at two higher conditions 320 °C (∼114.0 bar) and 340 °C (∼146.8 bar).

Reaction time after attaining desired temperature was always 30 min and continuous agitation of 100 rpm. The pressure of the reaction was not controlled and maintained as auto generated with respect to its reaction temperature.

The heating up time was recorded based on the selected temperature, ranging between 1 h and 3 h. After achievement of each batch experiment, the reactor was cooled down in a room temperature water bath (∼25 °C) until going back to its initial condition.

### 2.5 Cleaning of the reactor and separation of products

Fig. 1, also presents the schematic diagram of the experimental separation procedures after hydrothermal liquefaction of primary sludge. The products obtained are distributed into 4 different phases: gas, organic, aqueous, and solid.

When the reactor was back to atmospheric pressure and laboratory ambient temperature, the gas phase was released. The output gas was passed through a flow meter, indicating the volume of the gas mixture, and collected in a gas bag. After that, the reactor was opened, and the mixture was poured into a large beaker.

The solid part was separated from the liquid part via vacuum filtration. The liquid part, mainly containing the aqueous phase and a small part of the organic phase, was transferred into a bottle. Meanwhile, the reactor was washed repeatedly with dichloromethane until being totally clean to recover the organic remaining part, deposited on the walls, on the cover of the reactor and, in the agitation module. Then the mixture, where a part of solids was entrapped into the organics, was separated by vacuum filtration.

The liquid part containing the organic phase and the dichloromethane was transferred into another bottle. The solid retained on the filter paper, biochar, and ashes, was washed with dichloromethane several times. The biochar was dried in the oven for 24 h at 105 °C and quantified by weighting. A small volume of dichloromethane was added to the aqueous phase.

Then, the mixture was centrifuged at 8000 rpm for 5 min. The upper phase is the dichloromethane containing the small part of organic phase. This was added to the organic phase previously separated. The lower phase is the aqueous phase containing soluble organic molecules. Dichloromethane was evaporated from the organic phase by the rotary evaporation, at 65 °C and atmospheric pressure. The viscous organic liquid obtained is the biocrude, that it was further weighted for quantification.

Finally, biocrude was separated into oils and asphaltenes by Soxhlet extraction using 200 mL of hexane. Oils were separated from hexane by rotary evaporation at 65 °C and atmospheric pressure. Asphaltenes were quantified by the difference between biocrude and oils.

### 2.6 Products yields calculation

The biocrude yield was calculated from Equation (1):

(1) $$\text{Biocrude}\;\text{yield}\;\left(\%\right)=\frac{\text{Mass}\;\text{of}\;\text{biocrude}}{\text{Mass}\;\text{of}\;\text{volatile}\;\text{solids}}\times 100$$

The aqueous phase yield was calculated from Equation (2):

(2) $$\text{Aqueous}\;\text{phase}\;\text{yield}\;\left(\%\right)=\frac{\text{Mass}\;\text{of}\;\text{solids}\;\text{dissolved}\;\text{in}\;\text{aqueous}\;\text{phase}}{\text{Mass}\;\text{of}\;\text{volatile}\;\text{solids}}\times 100$$

The solid yield was calculated from Equation (3):

(3) $$\text{Solid}\;\text{yield}\;\left(\%\right)=\frac{\text{Mass}\;\text{of}\;\text{solid}\;\text{residue}}{\text{Mass}\;\text{of}\;\text{volatile}\;\text{solids}}\times 100$$

The gas yield was calculated from Equation (4):

(4) $$\text{Gas}\;\text{yield}\;\left(\%\right)=\frac{\text{Mass}\;\text{of}\;\text{gas}}{\text{Mass}\;\text{of}\;\text{volatile}\;\text{solids}}\times 100$$

In all the equations, mass of volatile solids is referred to that of primary sludge.

In the above expressions, mass of volatile solids is referred to that of primary sludge. Also, the yield of each phase is denoted to the average yield of three results obtained, accompanied with a standard deviation for error detection.

### 2.7 Products fine characterization

Fig. 1 again, shows the characterisation performed with the biocrude, which was very extensive: weight, ashes, gas chromatography/mass spectrometry (GC/MS), thermogravimetric analysis (TGA), elemental (ultimate) analysis, determination of higher heating value (HHV), Fourier transform infrared spectroscopy (FTIR), nuclear magnetic resonance (NMR) and simulated distillation (SimDis). Finally, saturated, aromatics, resins and asphaltenes (SARA) fractions of biocrude were characterised.

#### 2.7.1 The response surface of biocrude

The response surface of biocrude from HTL of primary sludge was evaluated through ANOVA. F-value test served as the primary means to compare the model variance the error variance associated with errors. A higher F-value indicates greater significance of the model. Model terms are considered significant if their associated P-values are less than 0.05 [25]. Each experiment has been carried out at least three times.

#### 2.7.2 Gas chromatography/mass spectrometry spectroscopy of biocrude

The samples of biocrude at all temperatures were characterized by gas chromatography-mass spectroscopy (GC/MS) using a PerkinElmer Turbo Mass Gold GC/MS, equipped with a Supelco SLB®-5 ms capillary GC column (L × I.D. 30 m × 0.25 mm, d_f_ 0.25 μm). Dichloromethane was used as solvent. The GC oven was maintained at 70 °C for 1 min, heated to 180 °C at a rate of 7 °C/min, then heated to 240 °C at a rate of 12 °C/min and finally 7 min hold at 330 °C.

#### 2.7.3 Thermogravimetric analysis of biocrude

The weight loss properties of biocrudes were studied by thermogravimetric analysis (TGA). In each test, about 3–4 mg of sample was heated from 30 °C to 800 °C at a nitrogen flow of 60 mL/min and a 10 K/min heating rate.

#### 2.7.4 Elemental analysis and HHV of biocrude

Ultimate analysis of biocrude samples was also realized by Serveis Tècnics de Recerca at Universitat de Girona as commented in section 2.2. C, H and N were quantified, and O calculated by difference. Then, the higher heating values of (HHVs) of biocrude were calculated using Dulong formula (5), taken from Hong's study, where HHV is expressed in MJ/kg [26]:

(5) $$\text{HHV}\;\left(\text{MJ}/\text{kg}\right)=0.3383\cdot C+1.443\cdot \left(H-\frac{O}{8}\right)$$

C, H and O are the mass percentages of carbon, hydrogen, and oxygen from the ultimate analysis of the samples, respectively.

#### 2.7.5 FTIR of biocrude

FTIR spectra were collected using a Thermo Nicolet Nexus 670 Fourier Transform Infrared Spectrophotometer equipped with a single-bounce diamond attenuated total reflectance (ATR) accessory (Specac Golden Gate) and KBr beam splitter. Spectra were collected from 4000 to 500 cm^−1^ with 0.98-cm^−1^ resolution and averaged over 50 replicate scans using Omnic software. Background scans were conducted of the dry accessory at ambient temperature. The spectra were then collected after smearing about 30 mg of sample directly on the ATR crystal surface.

#### 2.7.6 ^1^H NMR of biocrude

^1^H NMR spectra were collected using a Varian Unity 400-MHz spectrometer outfitted with a 5-mm broadband probe. 50–75 mg of biocrude were dissolved in deuterated chloroform containing 0.03% tetramethyl silane (TMS) as an internal reference. Samples were then filtered (0.22-lm PTFE) to remove any suspended particulates before loading into 5 mm diameter NMR tubes. ^1^H spectra were acquired with a 90° pulse angle, spinner frequency of 20 Hz, sweep width of 8000 Hz across 32 transients.

#### 2.7.7 Simulated distillation of biocrude

Simulated distillations were modelled after ASTM-D2887 method and performed using a HP 5890 Series II FID gas chromato-graph and a Durabond DB-HT-SimDis GC column by Agilent-J&WScientific (5 m0.53 mm id, 0.15 μm film). Helium (56.4 mL/min) was used as the carrier gas. The oven temperature was initially set to 36 °C, and raised to 400 °C at 10 °C/min and then held constant for 10 min. The injector volume was set to 0.5 μL and the injector temperature was set to 350 °C. Detector temperature was set to 375 °C, hydrogen gas set to 40 ml/min, airflow set to 400 ml/min, and helium makeup set to 24 ml/min. Samples (1% w/w) and reference standards (0.5% w/w) were dissolved in DCM. Samples were filtered (0.22 μm PTFE) to remove any suspended particulates. Boiling points were determined in accordance to a D2887 calibration mix and a D2887 Reference Gas Oil standard, both purchased from sigma Aldrich. Data (retention time and areas) were collected. Each sample was distributed between fractions (% w/w) and boiling points were calculated accordingly.

#### 2.7.8 Gas chromatography/mass spectroscopy of oils separated from biocrude

Oils were also characterized by gas chromatography-mass spectroscopy (GC/MS). The same procedure than for biocrude was used (see section 2.6.1.), but hexane was utilized as solvent.

#### 2.7.9 Quantification of SARA fractions of biocrude

SARA fractions of biocrude were analysed. The separation of light phase and heavy phase was repeated as mentioned above, but with n-heptane. The separated maltenes were fractionated into saturated hydrocarbons with 20 mL of n-heptane using activated alumina in a glass chromatographic column. Then, aromatic compounds were extracted through 20 mL of toluene. Finally, resins were removed from the adsorbent using 20 mL of a mixture of toluene and 2-propanol (1:1). More polars were also removed using 20 ml of methanol. Each eluted fraction was recovered by solvent removal using a Rotary evaporator.

### 2.8 Biochar characterization and quantification

Fig. 1 too, presents the characterisation performed with the biochar. Total solids, moisture content, volatile solids and ash content were determined in biochar according to standard methods 2540B and 2540E respectively [22]. Also, ultimate analysis and heavy metals detection were done by following the same procedures described above.

### 2.9 Aqueous phase characterization

Fig. 1 as well, shows the characterisation performed with the aqueous phase. COD, TOC, TN, proteins, and carbohydrates were measured or analysed for the aqueous phase. COD analysis was performed according to standard method 5220D [22]. TOC was analysed by using a TOC analyser TOC-L Series based on a specific standard calibration curve. Total organic carbon (TOC) was measured by ASI-L auto sampler Shimadzu into a Shimazdu TOC-L CSN TOC analyser provided with a NDIR detector and calibrated with standard solutions of hydrogen potassium phthalate. Total dissolved nitrogen was measured in the same TOC analyser coupled with TNM-L ROHS unit [27].

Protein amount was measured according to Lowry method [24] and carbohydrates were quantified following Dubois method [23] as described in the previous section (2.3). Total solid (TS), volatile solid (VS) and ash content were measured in the aqueous phase as well. A specific volume of aqueous phase was dried in a weighted crucible for 24 h in the oven at 100 °C then burned in the furnace at 550 °C for 1 h, as detailed by the standard methods 2540B and 2540E respectively [22].

Measurement of pH value in the HTL aqueous phase was performed by pH meter. Heavy metals were analysed in the ash of the solid dissolved in the aqueous phase by following the same procedure mentioned before.

### 2.10 Gas phase characterization

Fig. 1 likewise, shows the characterisation performed with the gas phase. Identification and quantification of biogas were finalized by a gas chromatograph (micro-GC, Agilent, 990) equipped with a thermal conductivity detector (TCD). A MS5A SS 10M × 0.25MM × 30UM BF RTS, CP-PORABOND Q 5MX0.25MMX3UM column (column 1) was used to separate the light gases using Argon as a carrier gas and a PORAPLOT Q UM 10MX0.25MMX8UM BF, CP-PORABOND Q 1MX0.25MMX3UM column (column 2) was used to separate heavy gases using helium as a carrier gas. Column 1 was maintained at injector temperature 100 °C, injection time 40 ms, column temperature 100 °C and initial pressure 200 kPa. Column 2 was maintained at injector temperature 100 °C, injection time 40 ms, column temperature 60 °C and initial pressure 150 kPa. The run time was 120 s. The mole percentage of each gas was determined with respect to gas standards prepared by Carburos Metálicos, S.A.

## 3 Results and discussion

### 3.1 Characterization of primary sludge and suitability of its use in HTL

The characterisation of primary sludge is outlined in Table 1. As it can be seen in the table, the primary sludge, as received, contained 4.3 ± 0.1% of total solids (w/w wet sludge basis). The moisture, calculated by difference, accounted to 95.7 ± 0.1% (w/w wet sludge basis). These values are close to those obtained in other studies of our research group with primary sludge from the WWTP of Reus (Tarragona, Spain): 4.2 ± 1.2% [13] or 3.9 ± 0.1% [14].

**Table 1:** Characterization of primary sludge from WWTP of Reus.

Feedstock characterization	Percentage %
Moisture content (w/w wet sludge basis)^a^	95.7 ± 0.1
Total solids (w/w wet sludge basis)^a^	4.3 ± 0.1
Volatile solids (w/w total solids basis)^a^	77.1 ± 0.3
Ashes in total solids (w/w total solids basis)^a^	22.9 ± 0.3
Proteins in volatile solids (w/w total solids basis)^a^	21.2 ± 1.7
Carbohydrates in volatile solids (w/w total solids basis)^a^	29.8 ± 1.2
Lipids in volatile solids (w/w total solids basis)^a^	23.4 ± 0.8
C	36.86
H	5.34
N	3.71
O^b^	31.19
Density of wet sludge (g/mL)	1.01
TOC in wet sludge (mg/L)	6290
HHV of wet sludge (MJ/kg)	14.55
Mass balance (Ashes + Proteins + Carbohydrates + Lipids)^a^	97.3 ± 4.0

a Average of at least three assays.

b By difference taking into account the quantity of ashes.

Ashes were 22.9 ± 0.3% (w/w total solids basis) of the total solids. Then, volatile solids were calculated by difference from ashes, 77.1 ± 0.3% (w/w total solids basis). On the other hand, the density of primary sludge was 1.012 g/mL, comparable to that of water. The volatile solids were analysed for carbohydrates, proteins, and lipids contents. Carbohydrates was found to be the predominant fraction (29.84%). Indeed, the values of lipids (oil, greases, fats, and long fatty acids) (23.41%) and proteins (21.15%) were considered significant. These results are comparable with those obtained in previous studies with the primary sludge of the same origin: lipids (19.6 ± 0.6%), carbohydrates (31.3 ± 0.1%), proteins (27.7 ± 0.1%) and ashes (16.0 ± 0.1%), values obtained in a study of recovery of cellulose from primary sludge [14] or, lipids (27.2 ± 0.4%), carbohydrates (26.2 ± 2.6%), proteins (24.2 ± 1.4%) and ashes (20.1 ± 0.4%), results from a primary sludge used to produce biodiesel. One of the characteristics in the total solids of the primary sludge of the Reus WWTP is the similar composition of the ingredients, all always between 20 and 30% in w/w total solids basis.

The ultimate analysis gave the following results: primary sludge had low nitrogen content (3.71%), low hydrogen content (5.34%), high carbon content (36.86%) and finally, a very high oxygen content (31.19%), this last one, obtained by difference. These values allowed to calculate the higher heating value (HHV) of dried primary sludge. In the calculation, the mass of the ashes has been discounted to obtain a more realistic value. In these conditions the HHV was 14.55 MJ/kg. This value is comparable with that obtained in other works: 10.55 MJ/kg [20] or 17.31 MJ/kg [28].

SEM images and EDX spectra of ash in primary sludge are presented in SI1 (Supplementary information). SEM images show that the ashes have an irregular structure. The particles have different size and are irregular. It seems that the bigger particles are formed by aggregation of smaller ones. The image magnified 6000 times (location (c)) shows asymmetric cavities and a surface full of granules. In the case of metals contained in the ashes, EDX spectra identified in average: O (41.2%), Ca (17.1%), Fe (13.4%), Si (7.0%), P (5.3%), Al (4.3%), S (2.9%), Cl (1.7%), Na (1.6%), K (1.3%), Mg (1.2%) and Ti (0.7%). The origin of these oxides should be the drinking water that has high calcium content, the dust dragged by rainwater and the erosion caused in the sewage system.

Concerning the suitability to use primary sludge in HTL process, the value of total solids in the primary sludge is low, 4.3% (w/w wet sludge basis). This value is consistent with the values obtained in other works, which usually vary between 1 and 5%, such as 5.0% (Biller et al., 2018) or 4.5% [21]. This means that the water content present in the sludge is very high. As it was commented in introduction, HTL process is a very good option to treat wet biomass, where water is used as the medium for breaking down the organic matter.

Lipids in primary sludge are formed from free fatty acids in the range of C10 to C18 which are precursors for esters production. Also, proteins are approved to be promoters for biocrude production through HTL. Maillard reactions represent an important part in the distribution of biocrude and composition, originated from the reaction of amine groups present in proteins with carbonyl groups present in reducing carbohydrates [2]. WWTP primary sludge is rich in lipids, proteins and carbohydrates. Therefore, HTL is assumed to be a suitable option for thermally hydrolysing the macromolecules into valuable chemicals. Then, primary sludge assists in demonstrating an economically viable and energy-efficient sludge biorefinery approach.

### 3.2 Hydrothermal liquefaction of primary sludge

HTL experiments of primary sludge, sample around 500 g, were always performed at a reaction time of 30 min and a stirring rate of 100 rpm. Five different operating temperatures were utilized: 240, 270, 300, 320 and 340 °C. After the completion of the reaction time, the reactor was cooled down to ambient conditions and the four products (gaseous phase, biocrude, containing organics aqueous phase and biochar) were separated following experimental procedure depicted in Fig. 1. The quantification and characterization of the products obtained during the HTL of the sludge will be presented in the different sections presented below.

### 3.3 Conversion of primary sludge to biocrude

#### 3.3.1 Results

The biocrude yields at different temperatures are illustrated in Table 2.

**Table 2:** Biocrude, oils and asphaltenes yields after HTL experiment, 30 min of reaction time and 100 rpm stirring rate.

	Biocrude		Oils^a^		Asphaltenes^a^	
T (°C)	Weight (g)	(%)	Weight (g)	(%)	Weight (g)	(%)
240	5.86 ± 0.35	35.17 ± 2.03	3.47 ± 0.68	22.67 ± 0.80	2.10 ± 0.52	12.50 ± 0.47
270	6.66 ± 0.12	39.47 ± 0.74	4.02 ± 0.06	23.87 ± 0.40	2.64 ± 0.18	15.60 ± 0.34
300	6.29 ± 0.13	37.66 ± 0.66	4.08 ± 0.16	23.96 ± 0.71	2.21 ± 0.26	13.70 ± 1.63
320	6.11 ± 0.20	37.42 ± 0.33	3.69 ± 0.32	21.24 ± 0.89	2.42 ± 0.51	16.18 ± 1.34
340	5.82 ± 0.15	34.97 ± 0.28	2.92 ± 0.22	17.47 ± 0.92	2.89 ± 0.22	17.50 ± 0.60

a Oils and asphaltenes were obtained from biocrude by separation with hexane.

Overall, the biocrude yield initially increased and then decreased with temperature increasing from 270 to 340 °C. The maximum value was obtained at 270 °C, 39.47% (w/w_VS_). This value is consistent with other works, where a close value of 42.20% (w/w_VS_) was obtained in the HTL of primary sludge at 330 °C for 10 min [29], and comparable to that obtained with HTL of municipal sludge, 34.00% (w/w_VS_), at 325 °C for 30 min [30].

A slight drop, ∼2.0% (w/w_VS_), from 300 °C to 340 °C should be caused by gas formation through biocrude conversion. Similarly, temperature also has a critical effect on the distribution of biocrude between oils and asphaltenes. Herein, the maximum value of oils, 23.96% (w/w_VS_), was reached at 300 °C. At 320 °C, oils yield has faced a sharp decrease to 21.24% (w/w_VS_). The results obtained confirm the significant effect of temperature on the biocrude yield. In other words, an important biocrude yield is supported by a high reaction temperature [1,31,32]. As mentioned elsewhere, higher temperature could boost the energy needed to break the bonds that strengthen hydrolysis and depolymerization of high-volatile biomass (carbohydrates, proteins, and lipids) [4]. Nevertheless, an increase in temperature over the optimal limit develops a thermal cracking of biocrude that reduce the biocrude yield and promotes the formation of coke and water-soluble gas products [33]. After passing the threshold value, the liquid intermediates are broken down into lighter fragments, that might be lost during the evaporation process of solvent [2]. Regarding the results obtained from SimDis analysis (Table 7), more jet fuel fractions were observed at high conditions. Also, this was validated by the decrease of biocrude yield when the temperature became higher than 270 °C, or by the apparition of ethylene and propene in the gaseous phase.

#### 3.3.2 ANOVA for biocrude yield

The response surface of biocrude yield from primary sludge was projected using ANOVA. Table 3 presents ANOVA results.

**Table 3:** ANOVA of biocrude yield from HTL experiments, 30 min of reaction time and 100 rpm stirring rate.

Source of Variation	Sum square	Mean square	F-value	P-value
Temperature	44.14	11.04	11.31	0.000688

Based on the assessment of P-value and F-value, temperature contributes importantly to biocrude yield. P-value was 0.000688, which is lower than 0.05 and F-value was 11.31, which is considered high. In another study, the results obtained from the ANOVA of biocrude yield from HTL of bagasse demonstrated that the reaction temperature is a significant factor on the biocrude yield as they obtained a P-value of 0.0259 and a F-value of 11.9 [35].

#### 3.3.3 Ultimate analysis and HHV of biocrude

The ultimate composition C, H, N and O, atomic ratios, higher heating values HHV and the energy recovery ER of biocrude are presented in Table 4. Also, the properties of petroleum are listed as well to perceive the quality of biocrude [34]. The ultimate composition of biocrude was not really affected by the effect of temperature, because results are close. The percentages of C and O reacted with temperature in an opposite way. We can notice that the carbon content in biocrude kept increasing with temperature until reaching an optimum value of 76.94 % at 340 °C.

**Table 4:** Ultimate analysis and HHV of biocrude, 30 min of reaction time and 100 rpm stirring rate.

Samples		% C	% H	% N	% O	H/C	O/C	HHV (MJ/kg)*	ER (%)
Primary S.		47.81	6.93	4.81	40.45	2.03	0.74	18.87^#^	–
Biocrude	240 °C	74.89	11.59	3.65	9.87	1.86	0.10	40.28	75
	270 °C	75.11	10.81	4.38	9.69	1.73	0.10	39.26	82
	300 °C	75.06	11.31	4.07	9.56	1.81	0.10	39.99	73
	320 °C	75.82	10.95	4.17	9.06	1.73	0.09	39.81	79
	340 °C	76.94	11.01	4.20	7.85	1.72	0.08	40.50	75
Petroleum^**@**^		83–87	10–14	0.1–1.0	0.1–3.0	–	–	∼ 42.75	–

* Calculated with Dulong equation [26].

^#^ Dried in the oven during 24 h at 105 °C.

^@^ [34].

Whereas, the oxygen content was generally in decline, from 9.87% to 7.85% at 340 °C. The nitrogen content values have been quite constant and have remained within the interval 3.65–4.38%. In the same way, the hydrogen content was rather constant for the five temperatures. Values are contained in the interval 10.81–11.59%. This last value was reached at 240 °C (11.59%). With these values, HHV was always high, around 40 MJ/kg.

**Fig. 2:**
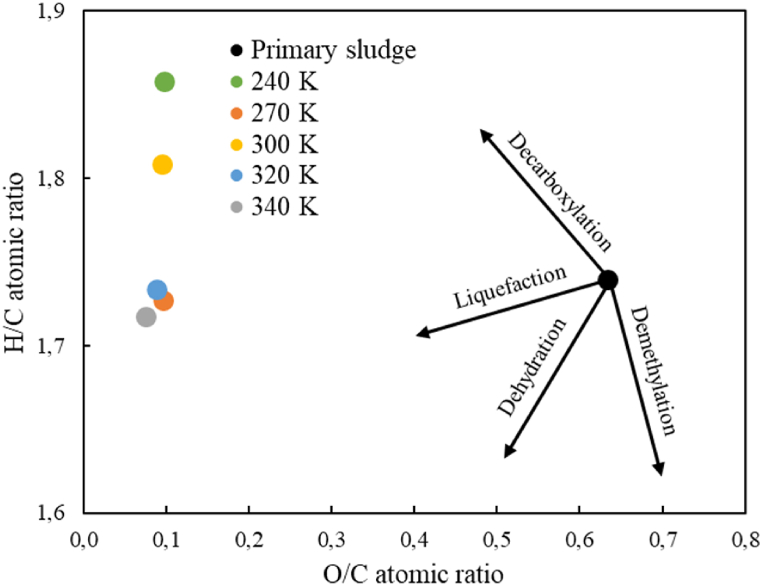
Van Krevelen diagram for primary sludge and biocrudes. 30 min of reaction time and 100 rpm stirring rate.

Comparing the results obtained to the one of petroleum biocrude, the value attained at 340 °C, 40.50 MJ/kg, was very close to the value of petroleum, 42.75 MJ/kg. On the other hand, the increase of HHV with respect to the original sludge (18.87 MJ/kg) is very important, close to 80% in all cases. On the other hand, the atomic ratios H/C and O/C have been calculated. The result of these calculations allows to represent the Van Krevelen diagram [36]. The diagram allows to evaluate the origin and the degree of maturity of the oil. It also allows visualizing the differences between this and other synthetic or biomass-derived fuels. Fig. 2 presents the Van Krevelen diagram for primary sludge and biocrude.

As can be seen in the figure, the O/C atomic ratio of biocrudes is much lower than that of primary sludge, there is a factor of one order of magnitude. This fact indicates that the transformation of primary sludge to biocrude has been carried out through dehydration and decarboxylation reactions, which allows the reduction of the O/C atomic ratio and therefore improves the stability and viscosity of the biocrude. Also, biocrude upgrading would require less hydrogen. On the contrary, the H/C atomic ratio of the primary sludge is very similar to that of the biocrudes. But on the other hand, this ratio is still a bit low compared to that of oil. This indicates that the H/C atomic ratio has to be improved by eliminating heteroatoms, as the only way for biocrude to be used as a substitute for petroleum-derived fuels. Even these results are encouraging, there is still a huge difference between the oxygen percentages in biocrude and fossil petroleum. The high content of O makes biocrude soluble in polar solvents including methanol and acetone, but badly mergeable with fossil fuels [31]. The target of the study is to obtain an intensification of energy density in biocrude to be qualified to be used in further applications, including biofuel. Therefore, upgrading biocrude is necessary to fit with the petroleum conditions. This can be done by either the introduction of catalysts, working under hydrogen environment, by the utilisation of an organic solvent or, a combination of all.

#### 3.3.4 GC/MS analysis of biocrude

Usually, there are more of 300 substances identified by GC-MS in the biocrude. The main substances identified from the ingredients of sludge as lipids, protein, carbohydrate and lignin, were cyclic terpanes and terpenes, along with nitrogenous, oxygenated, and phenolic components [37]. Chemical compounds of the biocrude obtained at different temperatures were identified by GC-MS analysis. The detailed compounds information of the biocrude from HTL at 300 °C are presented in Table SI1.

As it can be seen in the table, biocrude displayed various chemical groups. Oil compounds were mostly with chain structures and contained different number of nitrogen elements. Moreover, GC/MS results showed sufficient molecules in a cyclic form, suggesting the transform of hydrophilic molecule from sludge to the oil phase through recombination reactions [38]. On the other hand, more types of N-containing long-chain structure molecules were found in biocrude, implying the improved combination of alkane with amine generated from the deamination of the organics in the feedstock [39]. Phenols were conceivably produced from the cyclization/condensation of carbohydrates resulting from cellulose and hemicellulose component [40]. The chromatogram of biocrude at 300 °C is depicted in Figure SI2 (Supplementary information).

#### 3.3.5 TGA analysis of biocrude

**Fig. 3:**
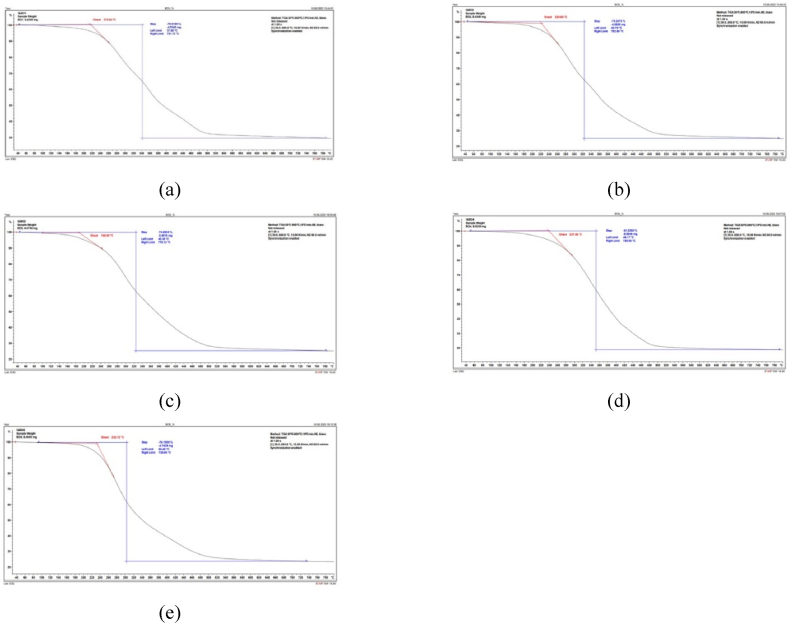
Thermal gravimetric analysis of samples of biocrude: (a) 240 °C; (b) 270 °C; (c) 300 °C; (d) 320 °C; (e) 340 °C. 30 min of reaction time and 100 rpm stirring rate.

TGA analysis was applied to study the three stages of weight loss of the biocrude. The TGA curves of the 5 samples of biocrude from HTL of primary sludge are shown in Fig. 3. All HTL biocrudes show the same TGA curve progression, with a similar decomposition process.

A relatively significant weight loss takes place at 215.04 °C in HTL-240 °C with 70.52% of weight loss, 220.66 °C in HTL-270 °C with 75.25 % of weight loss, 186.90 °C in HTL-300 °C with 74.69 % of weight loss, 237.56 °C in HTL-320 °C with 81.26 % of weight loss, 232.12 °C in HTL-340 °C with 76.15 % of weight loss.

#### 3.3.6 FTIR of biocrude

**Fig. 4:**
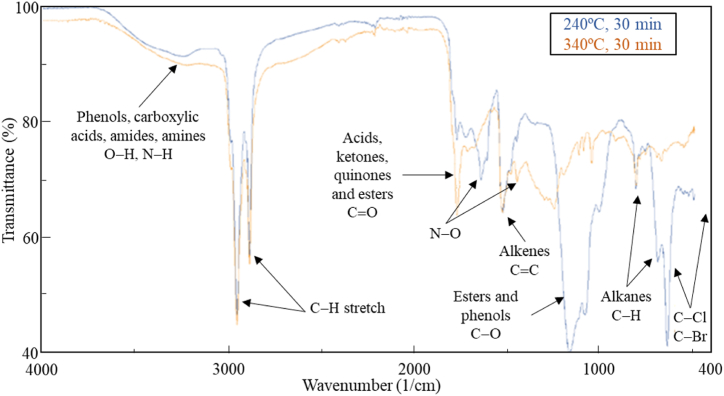
FT-IR plot of samples of biocrude: 340 °C (orange) and 240 °C (blue). 30 min of reaction time and 100 rpm stirring rate.

FTIR spectroscopy identifies functional groups present in biocrude and allows for a more comprehensive comparison of these groups when compared to GC/MS analysis. Fig. 4 shows FTIR spectra for biocrudes via hydrothermal liquefaction at 240 °C and 340 °C for the same reaction time (30 min).

Confirming ultimate analysis, the high hydrogen and carbon content of HTL biocrude at both conditions produced important saturated C-H stretching with CH_2_ and CH_3_ bending around 2919 cm^−1^ and 2850 cm^−1^, unsaturated stretching around 1657 cm^−1^ and aromatics around 720 cm^−1^. Biocrude was observed with C-O stretching peaks (1035–1456 cm^−1^) that belong to esters and phenols. C=O stretching peaks around 1707 cm^−1^ and 1780 cm^−1^ correspond to the functional groups of carboxylic acids, ketones, quinones and esters as expected from GC/MS chromatogram. The moderate proteins content was reflected in the biocrude with N-H bending peaks at 3195 cm^−1^ and 3215 cm^−1^. The peaks around 457 cm^−1^ and 403.3 cm^−1^ indicated the presence of C-Br or C-Cl bonds. Both spectra were very similar. They contained the same functional groups. However, the spectra of the HTL of biocrude at 340 °C detected more peaks that correspond to C=O functional groups, indicating the presence of wider variety of acids, ketones and esters.

#### 3.3.7 ^1^H NMR of biocrude

**Fig. 5:**
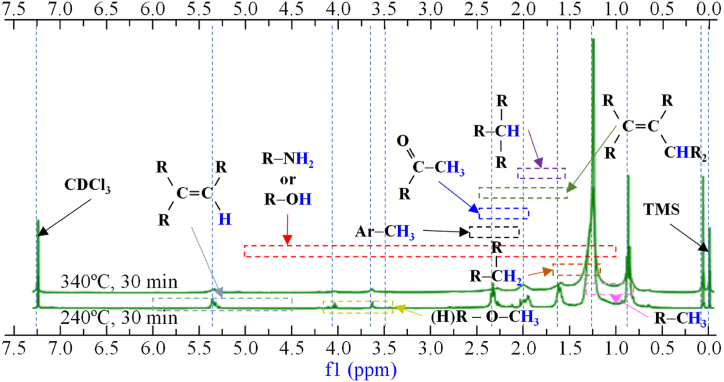
^1^H NMR plots of samples of biocrude at 240 and 340 °C. 30 min of reaction time and 100 rpm stirring rate.

NMR spectra provided complementary functional group information to FTIR spectra. Fig. 5 presents the ^1^H NMR spectra of samples of biocrude at 240 °C and 340 °C for the same reaction time (30 min).

Similar to FTIR, ^1^H NMR spectra showed a percentage of aliphatic functional groups of alkane functional groups (0.8–1.2 ppm). HTL biocrude exhibited unsaturated functionality (alkenes) (1.8–2.4 ppm). However, the same section can be assigned to carboxylic acids, ketones and esters. The high peak intensity here confirmed that most of the biocrude from HTL of sludge was contributed by the decomposition of lipids-derived compounds [41].

Aromatics were also observed (2.2–2.4 ppm) in agreement with findings from FTIR. The chemical shifts, located between 5.2 and 5.5 ppm, represented amide protons, that can be associated to the large number of nitrogen compounds. The big peak around 7.2 ppm corresponded to the solvent used. Even though both conditions contributed to the same functional groups, but their peak intensities were very distinct. It can be noted clearly that the peaks of the biocrude at HTL 340 °C were way higher than that of HTL 240 °C. It can be concluded that more compounds were produced when the temperature has increased. This was also confirmed by GC/MS and FTIR.

#### 3.3.8 Quantification of SARA fractions of biocrude

SARA fractions quantification was performed to all conditions and the results are presented in Table 5. The biocrude was distributed between maltenes including saturates, aromatics and resins, and asphaltenes.

**Table 5:** SARA fractions characterization of biocrude, 30 min of reaction time and 100 rpm stirring rate.

Temperature	Oils				% Asphaltenes
	% Oils	% Saturates	% Aromatics	% Resins	
240 °C	50.40	30.80	6.22	13.38	49.60
270 °C	45.25	30.14	4.50	10.61	54.75
300 °C	42.43	29.00	4.74	8.69	57.57
320 °C	37.23	25.59	4.94	6.70	62.77
340 °C	41.98	24.20	7.86	9.92	58.02

The amount of asphaltenes occupied almost 50 % of the total amount of biocrude obtained. At 320 °C, the percentage was the highest (62.80 %). The amount of saturates was predominant. Although it didn't change at low and mild conditions (≈30 %), a slight decrease was noted at high temperatures (25 %). Aromatics were just few. Their maximum attaint was even lower than 8 %. Polar compounds were observed in all biocrudes. They decreased with the increase of the temperature until reaching a minimum value of 6.70 % at 320 °C then increased again to 9.92 % at 340 °C. The results obtained from SARA analysis complied with the peaks detected by GC/MS. These results are presented in Table 6.

**Table 6:** GC/MS of Saturates, Aromatics and Resins of oils from biocrude, 270 °C of temperature, 30 min of reaction time and 100 rpm stirring rate.

Saturates	Dibutyl phthalate; Eicosane; Octadec-9-enoic acid; 7-Pentadecyne; 11-Tricosene; Tetracosane; Hexadecanoic acid, dodecyl ester
Aromatics	Cyclopentane, 1-ethyl-2-methyl-, *cis*-; Ethylbenzene; o-Xylene
Polars	Tetradecanoic acid; n-Hexadecanoic acid; Oleic Acid; Octadecanoic acid; Octadecanamide

#### 3.3.9 Simulated distillation of biocrude

**Fig. 6:**
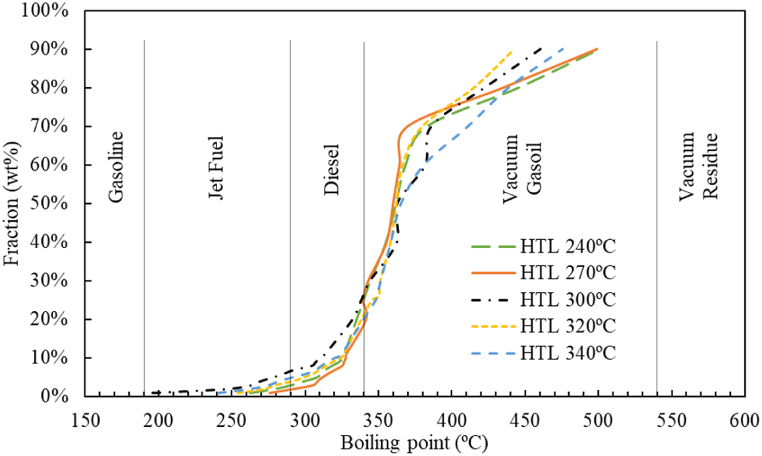
Simulated distillation of biocrude. 30 min of reaction time and 100 rpm stirring rate [42].

Fig. 6 shows the conversion of chromatograms from simulated distillation of biocrudes to their respective mass fractions depending on their boiling point. As it can be seen in the figure, the biocrudes produced at all temperatures offer very similar behaviours. The differences between the studied temperatures are not significant, being always below 10%. None of the five biocrudes have a fraction in the range of gasoline. Only less than 10% of the biocrude fraction is in the Jet Fuel range.

The substances included in this fraction are usually produced from protein and lignin [37]. Similarly, less than 30% of the biocrude fraction is in the Diesel range. The elements involved in this fraction are essentially formed from protein, and in minor quantity from lipids, carbohydrates and lignin [37]. In fact, 70% of the biocrude fractions are in the vacuum gasoil range. The substances involved in this fraction are essentially formed from lipids [37]. This fact demonstrates that biocrude requires further treatment in order to be considered as an alternative viable fuel for locomotion vehicles. As can be seen in other works [42], the use of biocrude to produce fuels requires refining after liquefaction. This post-refining must use commercial hydrotreating catalysts and hydrogen, both to improve the quality of the hydrocarbons and to eliminate the heteroatoms present in the biocrude mixtures. Table 7 displays the values of composition of biocrudes by fractional cuts. The table was made using the boiling cuts presented by Haider [42].

**Table 7:** Simulated distillation of biocrude. 30 min of reaction time and 100 rpm stirring rate.

Fractional cuts	BP range	Composition				
		240 °C	270 °C	300 °C	320 °C	340 °C
Gasoline	<190 °C	0%	0%	0%	0%	0%
Jet fuel	190-290 °C	3%	2%	3%	4%	5%
Diesel	290-340 °C	22%	25%	24%	17%	15%
Vacuum gasoil	340-538 °C	68%	68%	68%	73%	72%
Vacuum residue	>538 °C	7%	6%	5%	6%	8%

### 3.4 Aqueous liquid phase and its characterization

After the separation of organic phase from the liquid phase, aqueous phase was fully characterized. The liquid phase is essentially the moisture of primary sludge with water soluble organic species. The effects of temperature on the composition of aqueous phase are elucidated in Table 8. Apparently, with temperature rising from 240 to 300 °C, the TOC and COD concentrations have increased from 5130 to 5700 mg/L and from 10,130 to 15,700 mg/L. At 320 °C, the values of TOC and COD have decreased again to 5685 mg/L and 13,500 mg/L.

**Table 8:** Aqueous phase characterization after HTL.

Characterization	Temperature				
	240 °C	270 °C	300 °C	320 °C	340 °C
Weight (g)	427.98	447.00	451.14	398.01	438.09
Proteins (%, TS basis)	1.6	1.3	1.4	1.3	1.4
Carbohydrates (%, TS basis)	0.1	0.1	0.1	0.0	0.0
TOC (mg/L)	5130	5560	5700	5685	5440
COD (mg/L)	10,130	10,230	15,700	13,500	12,500
Density (mg/L)	1.00	1.00	1.00	1.00	1.00
pH	6.34	6.56	6.81	6.91	7.71
TS (%, dry basis)	0.86	0.66	0.42	0.45	0.32
Ash (%, TS basis)	13.7	17.2	22.5	24.0	29.6
VS (%, TS basis)	86.3	82.8	77.5	76.0	70.4

The much higher TOC and COD concentrations here suggests that large amounts of organic species in primary sludge are transferred into the aqueous phase during primary sludge HTL as dissolved organics. In all the scenarios, the value of COD is higher than 10,000 mg/L and the value of TOC is higher than 5000 mg/L. COD values are considered very high when compared to the one of the wastewater treatment influent (commonly 200–800 mg/L), which prevent its direct streaming back to the influent of WWTP [2].

The amounts of proteins and carbohydrates in the aqueous phase are very low, compared to the ones of primary sludge. HTL is carried out by hydrolytic breakage of bio-constituents of the wet biomass and by the improvement of the depolymerization products to simpler organic molecules. Here, by increasing the temperature, the fractions of proteins and carbohydrates in primary sludge were hydrolysed and transformed to organic products with simple chains. The density of aqueous phase is the same at all conditions and is comparable to the one of water. pH values were slightly increasing with temperature, indicating the existence of N-rich compounds. The percentage of dissolved solids in the aqueous phase was respectively low. Ash content and VS were changing oppositely with temperature. With the increase of temperature, the VS content in the aqueous phase was always dropping. Consequently, the ash percentage was increasing. SEM images and EDX spectra of ash in aqueous phase are presented in Figure SI2 (Supplementary information). Very little heavy metals remained in the ash of the dissolved solid in the aqueous phase. Several studies suggested the various technologies for the use of aqueous phase. Zhang and his co-workers have performed a supercritical gasification of aqueous phase at 700 °C produced H_2_ with 43.01 mol/kg of carbon [43]. While Shah and his co-workers have recirculated the aqueous phase and used it as a solvent in HTL. Energy recovery has increased in the form of biocrude by 50% [34].

### 3.5 Biochar and its characterization

#### 3.5.1 Experimental results

Biochar is the solid phase recovered after process. It's a black solid that contains carbonized organics and ashes. The results obtained from its analysis are presented in Table 9. The effect of temperature on the bio-char yield was always negative. When the temperature increased from 240 °C until 300 °C, the percentage of biochar decreased from 34.0 until 28.1% (w/w). However, when passing the threshold condition, the percentage of biochar was raised again. The calculated yields were 39.8% at 320 °C and 34.1% at 340 °C. At high temperatures, the biocrude was broken, favouring the production of higher amount of solid phase and gaseous products.

**Table 9:** Biochar characterization, 30 min of reaction time and 100 rpm stirring rate.

Characterization	Temperature (°C)				
	240	270	300	320	340
Biochar weight (g)	6.24	3.32	4.22	6.52	5.53
Biochar yield % (dry basis)	34.0	29.2	28.1	39.8	34.1
Ash % (dry basis)	33.2	58.4	60.6	62.93	76.8
VS % (dry basis)	66.8	41.6	39.4	37.07	23.2
C %	53.17	68.08	71.47	82.33	76.55
H %	7.22	8.13	9.75	11.84	10.13
N %	2.69	4.40	2.97	2.56	3.06
O %^a^	36.92	19.40	15.81	3.26	10.26
HHV (MJ/kg)	22.51	30.04	33.48	41.00	37.56

a By difference.

Ash and VS contents were dependent on the operating temperature. High temperature was promoting the amount of ash in biochar, resulting in lower VS. While the organic part in biochar was transferred to biocrude, the probability for its usage in further applications was decreasing. The solid residue, or biochar, is usually overlooked as a by-product despite the content of organic char and other nutrient elements. Actually, biochar ranging from 28.1% to 39.8% with a minimum VS of 23.2% and carbon percentage of 53.17% is considered a significant end product. It can be used as adsorbent as it presents a large surface area, porous structure to remedy the soils contaminated with heavy metals and organic compounds through adsorption [44].

#### 3.5.2 Ultimate analysis

The ultimate analysis of the solid phase, biochar, is also presented in Table 9. Solid phase from HTL of primary sludge could be implemented in different applications in various sectors due to its high energy density and high carbon percentage, especially at high conditions (320 and 340 °C). For example, solid phase reached a HHV of 41 MJ/kg with a carbon content of 82.33 %. Therefore, biochar can be considered a potential bioenergy feedstock. In addition, it can be used in different applications in soil amendment, storage material for hydrogen, catalysts for bioenergy conversion processes and construction materials [45]. Moreover, biochar can be utilized as well as biosorbent and carbon supplement in anaerobic digestion [46]. SEM images of biochar and ash in biochar are presented in Figure SI3 (Supplementary information).

#### 3.5.3 Heavy metals

EDX spectra of biochar and ash in biochar are also presented in Figure SI3 (Supplementary information). As carbon was the most abundant element in biochar, heavy metals were shown in small quantities. On the other hand, ash was rich in salts or oxides containing K, Ca, Mg, Fe, Na, S, Cl and Si. It was reported in some studies that heavy metals are accumulated in the solid residue from HTL using sewage sludge as feedstock [15].

### 3.6 Biogas

The volume and composition of produced biogas are presented in Table 10.

**Table 10:** Biogas composition, 30 min of reaction time and 100 rpm stirring rate.

Temperature (°C)	Biogas Volume (mL)	Biogas Composition % (mol fraction) *				
		CH_4_	CO	CO_2_	C_2_H_4_	C_3_H_6_
270	471.20	N.D.	N.D.	0.0465	0.0040	N.D.
300	857.67	0.0380	0.1150	0.0520	0.0040	N.D.
320	917.60	0.0350	0.0520	0.0480	0.0030	0.0020

* Values of H_2_O, N_2_, O_2_ are not shown.

N.D. Not detected.

In the table, nitrogen and oxygen have not been taken into account, whose peaks, especially the first, are the most important (see chromatograms in Figure SI4 of the supplementary information). Based on the results obtained from the gas analysis, and as it was expected, the increasing of the temperature from 270 to 320 °C, provoked the increase of the volume of gas produced, from 471.20 to 917.60 mL. The increase in temperature has also resulted in the production of more hydrocarbon molecules. At 270 °C only methane was detected, at 300 °C ethylene was added and finally at 320 °C propene was also detected. In fact, the composition of the other substances, carbon monoxide and carbon dioxide, are very stable in reference to temperature, the monoxide with a molar fraction between 0.05 and 0.10%, the dioxide with a very constant molar fraction close to 0.05%.

## 4 Conclusions

Hydrothermal liquefaction is an efficient valorisation process for wet residual biomass, like primary sludge. HTL allows the production of a noteworthy yield of biocrude with a great energy recovery value and some other valuable materials from WWTP primary sludge. The effect of temperature over conversion and quality of products was realized. The best production of biocrude, 39.47% (w/w_VS_), was achieved at 270 °C. HHV attained a value of 40.50 MJ/kg, close to oil value. The best value of the chemical energy recovered was 82% at 270 °C, and still 79% at 320 °C. The higher conversion to oils, 23.96% (w/w_VS_), was attained at 300 °C. The increase of temperature provoked in the biocrude the growth of the proportion of asphaltenes, heavier than oils. Nevertheless, the biocrude contains close to 10% of oxygen and 5% of nitrogen, which makes subsequent refining necessary if energy vectors are scheduled.

In the case of the other products, Aqueous phase still contains soluble organics, TOC over 5 g/L and COD over 10 g/L for all conditions, which makes it an interesting feedstock to produce biogas by hydrothermal gasification (HTG). Conversion to biochar is always higher than 20%. The higher HHV, 41 MJ/kg, was obtained at 320 °C. Close to 1 L of biogas was produced at 320 °C. Methane, ethylene and propene were identified in the biogas, with a molar fraction close to 1.5%.

ANOVA statistical analysis have confirmed the significant effect of temperature on biocrude yield, confirming trends observed in the experimental results.

These excellent results are caused by the high quantities of lipids and carbohydrates in the primary sludge. In definitive, HTL of primary sludge is a very attractive process where a residual biomass is converted in four products, all with industrial interest.

## Funding

Jacky Cheikhwafa thanks the 10.13039/501100003030Agència de Gestió d’Ajuts Universitaris i de Recerca (10.13039/501100003030AGAUR) of Catalan Government for the pre-doctoral contract (Ajuts per a la contractació de personal investigador predoctoral en formació, 2019 FI-B 00743). This work is part of the PECT “Cuidem el que ens uneix/Pobles Vius i Actius/Ebrebioterritori” project, within the frame of the RIS3CAT and ERDF Catalonia Operational Programme 2014–2020. It is co-financed by the Catalan Government and the Provincial Council of Tarragona. Work was co-financed by the 10.13039/501100007512Universitat Rovira i Virgili (2017PFR-URV-B2-33).

## CRediT authorship contribution statement

**Jacky Cheikhwafa:** Conceptualization, Data curation, Formal analysis, Investigation, Methodology, Writing – original draft, Writing – review & editing. **Katarzyna Glinska:** Formal analysis, Investigation, Methodology. **Esther Torrens:** Conceptualization, Funding acquisition, Project administration, Resources, Supervision. **Christophe Bengoa:** Conceptualization, Data curation, Funding acquisition, Methodology, Resources, Supervision, Validation, Writing – original draft, Writing – review & editing.

## Declaration of competing interest

The authors declare that they have no known competing financial interests or personal relationships that could have appeared to influence the work reported in this paper.
