# Supplementary material for: Effect of temperature on hydrothermal liquefaction of high lipids and carbohydrates content municipal primary sludge
Source: Heliyon. 2024 Jan 19;10(3):e24731. doi: 10.1016/j.heliyon.2024.e24731 (PMC10838732; doi:10.1016/j.heliyon.2024.e24731)

**Supplementary Information (SI)**

**Table IS1**. Substances identified in the chromatograms of biocrude at different temperatures, 30 min of reaction time and 100 rpm stirring rate. 30 min of reaction time and 100 rpm stirring rate.

| Formula | Substance | Time (min) | Temperature (ºC) | | | | |
| --- | --- | --- | --- | --- | --- | --- | --- |
|  |  |  | 240 | 270 | 300 | 320 | 340 |
| C_18_H_36_* | 1-Octadecene or 5-Octadecene | 13.92 |  | X |  |  |  |
| C_16_H_32_ | Cetene | 14.94 |  | X | X |  |  |
| C_16_H_34_ | Hexadecane | 15.70 |  | X |  |  |  |
| C_14_H_21_NO | Ethyl N-isopropyl-3-phenylpropanimidate | 16.53 |  |  | X |  |  |
| C_12_H_22_ | 1,11-Dodecadiene | 17.01 |  |  |  |  | X |
| C_16_H_30_O_2_ | E-14-Hexadecenal | 17.24 |  |  |  |  | X |
| C_17_H_34_ | 1-Heptadecene | 17.27 |  |  | X |  |  |
| C_12_H_24_O | 2-Dodecanone | 17.41 |  |  | X |  |  |
| C_15_H_30_O | 2-Pentadecanone | 17.41 | X | X |  |  | X |
| C_14_H_28_O_2_ | Tetradecanoic acid | 18.30 |  |  | X |  | X |
| C_18_H_38_ | Octadecane | 18.75 | X | X | X |  | X |
| C_12_H_24_O_3_* | 3-hydroxy-Dodecanoic acid | 18.88 |  |  |  |  | X |
| C_18_H_26_O | Octadecanone | 19.58 |  |  | X |  | X |
| C_20_H_38_O_2_ | Paullinic acid | 19.63 |  |  |  |  | X |
| C_13_H_24_O | Cyclotridecanone | 19.83 |  |  |  |  | X |
| C_19_H_40_ | Nonadecane | 19.96 |  |  |  |  | X |
| C_10_H_22_ | Decane | 19.96 |  |  | X |  |  |
| C_17_H_34_O | 2-Heptadecanone | 20.02 | X |  |  |  | X |
| C_14_H_28_O_2_ | Tetradecanoic or Myristic acid | 20.27 |  |  |  |  | X |
| C_17_H_34_O_2_ | Heptadecanoic acid | 20.27 | X | X | X | X | X |
| C_10_H_20_ | 1-Decene | 20.56 | X |  |  |  |  |
| C_16_H_32_O_2_* | n-Hexadecanoic acid | 20.68 | X | X | X |  |  |
| C_20_H_42_ | Eicosane | 21.02 |  |  |  |  | X |
| C_19_H_38_* | 1-Nonadecene | 21.13 |  |  | X | X |  |
| C_34_H_70_ | Tetratriacontane | 21.96 |  |  |  |  | X |
| C_20_H_41_Cl | 1-Chloroeicosane | 21.97 |  | X |  |  |  |
| C_16_H_30_O_2_* | Palmitoleic acid or Hexadecenoic acid | 22.13 | X | X | X |  | X |
| C_18_H_34_O_2_ | Oleic or 6-Octadecenoic acid | 22.38 | X | X | X | X | X |
| C_18_H_34_O* | 2-Tetradecylcyclobutanone or 5-Octadecenal | 22.39 |  | X |  | X |  |
| C_18_H_36_O | Octadecanone | 22.58 |  | X |  | X |  |
| C_16_H_33_NO | Hexadecanamide | 22.77 |  | X | X | X |  |
| C_14_H_29_NO | Tetradecanamide | 22.78 |  |  |  |  | X |
| C_12_H_25_NO* | Dodecanamide | 22.78 | X |  | X |  | X |
| C_18_H_35_NO* | (9Z)-Octadecenamide | 24.26 | X | X | X | X | X |
| C_13_H_26_O | 2-Tridecanone | 25.85 |  |  | X |  |  |
| C_17_H_30_NO | Tridecanoic acid, pyrrolidide | 25.86 |  | X |  | X |  |
| C_27_H_46_ | Cholestenes | 27.40 |  | X | X | X | X |
| C_28_H_56_O_2_ | Octacosanoic acid | 28.21 | X | X | X | X |  |
| C_15_H_23_NO_3_ | p-Nonyloxynitrobenzene | 28.64 |  |  | X |  |  |

* Also observed in Shah et al., 2020

**Figure SI1.** SEM images and EDX spectra of ash in primary sludge. (a), (b) and (c) represent three different observation locations of the sample.

| 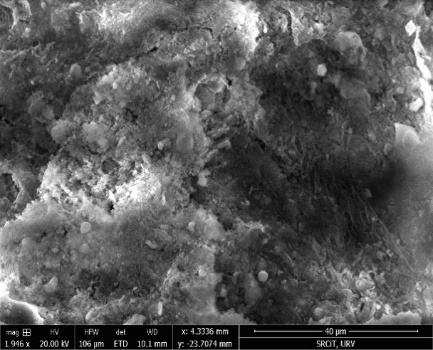  SEM image location (a) | 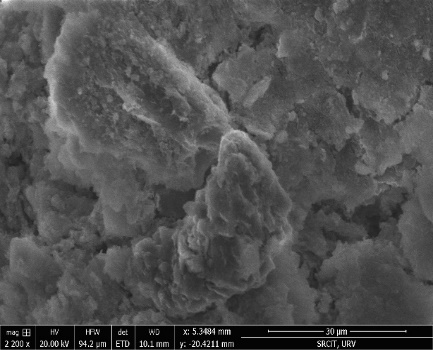  SEM image location (b) | 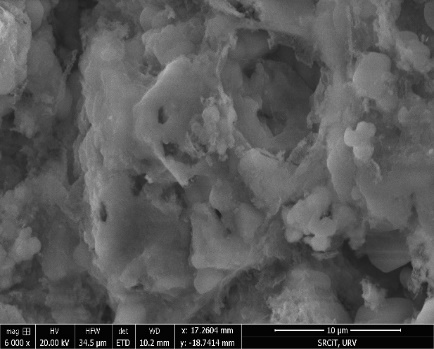  SEM image location (c) |
| --- | --- | --- |
| 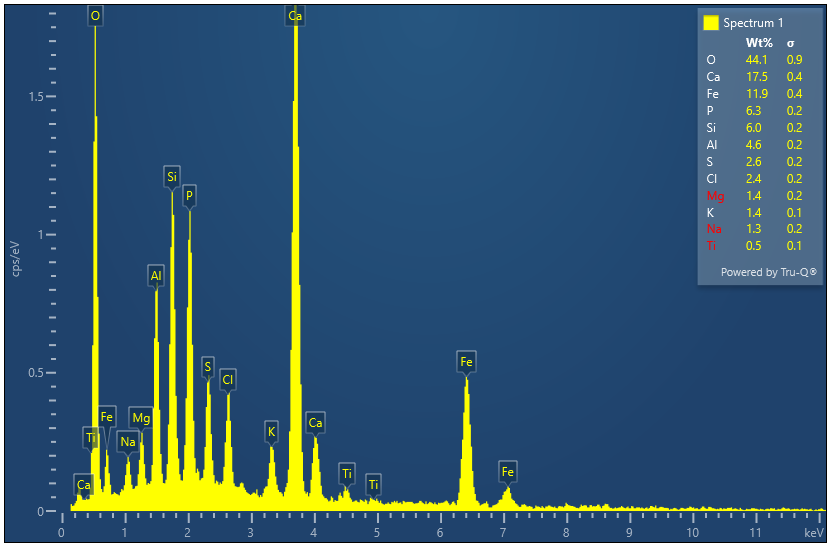  EDX spectra location (a) | 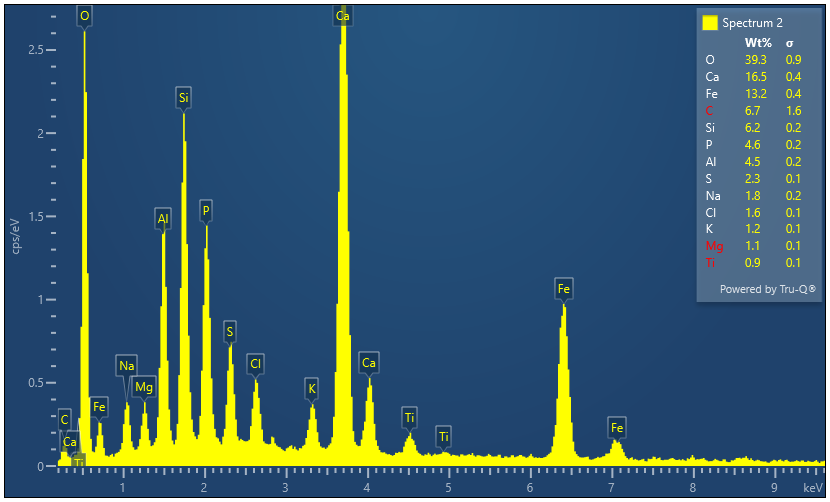  EDX spectra location (b) | 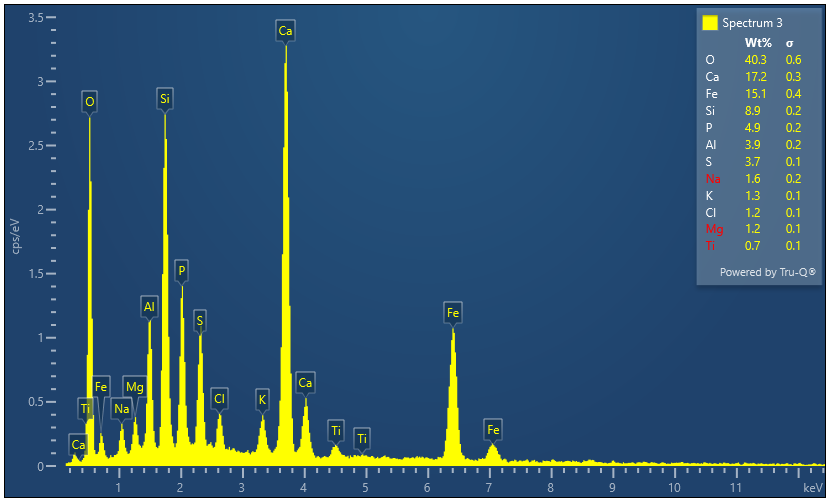  EDX spectra location (c) |

**Figure SI2**. Chromatograms of biocrude (a), saturated oils (b), aromatic oils (c) and resins (d). 300ºC, 30 min of reaction time and 100 rpm stirring rate.

| 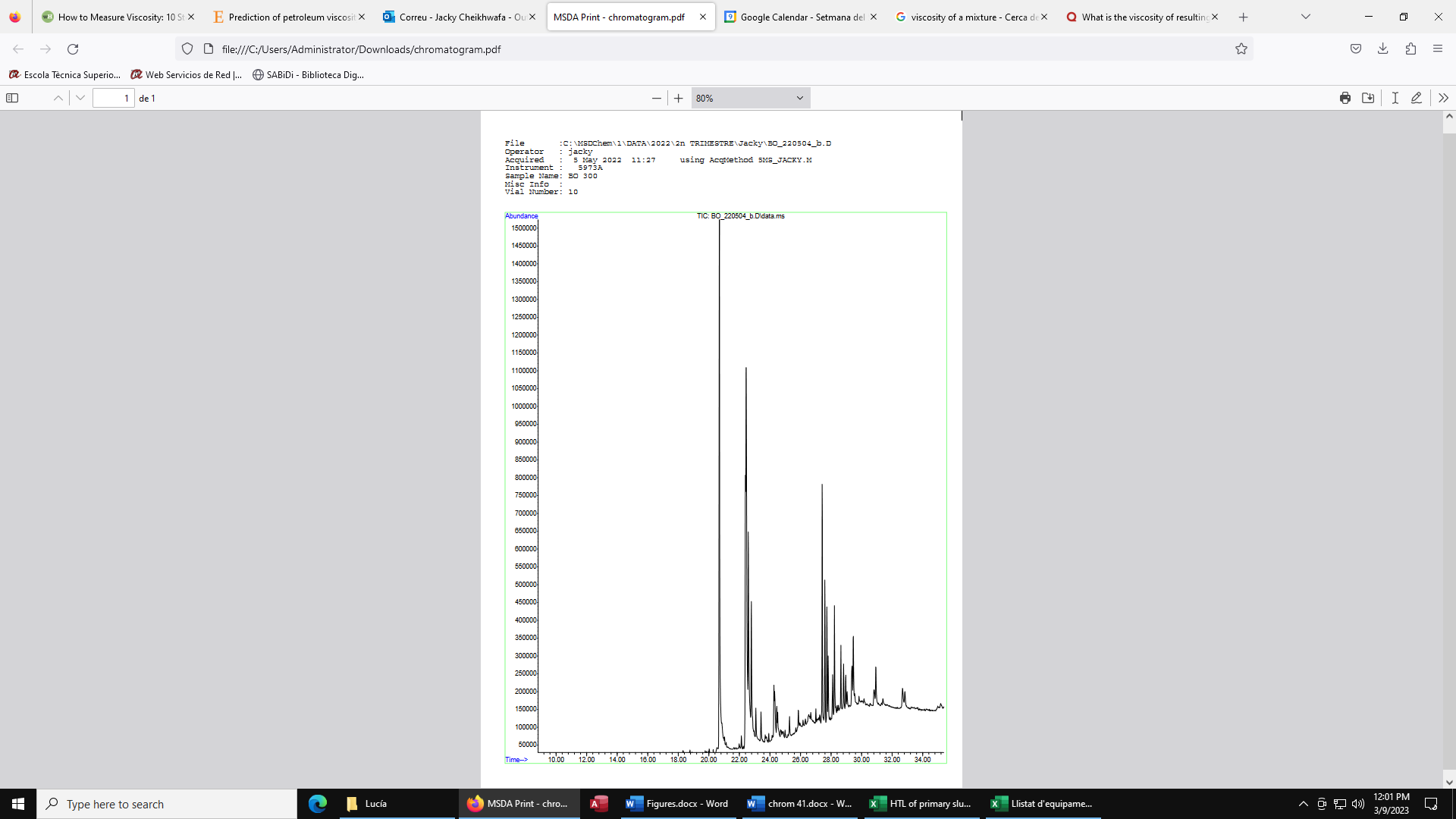 | 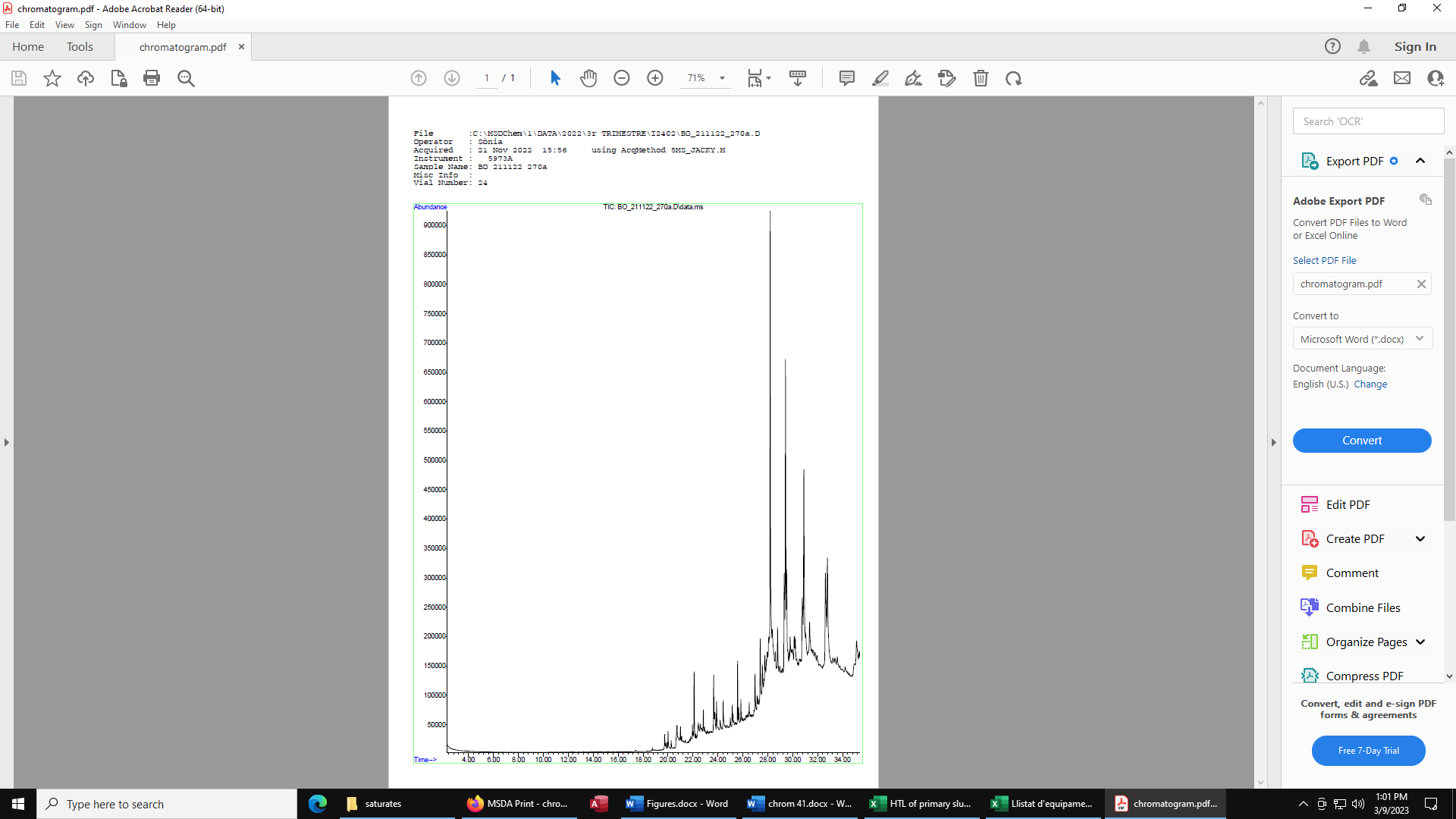 |
| --- | --- |
| (a) Biocrude | (b) Saturated oils |
| 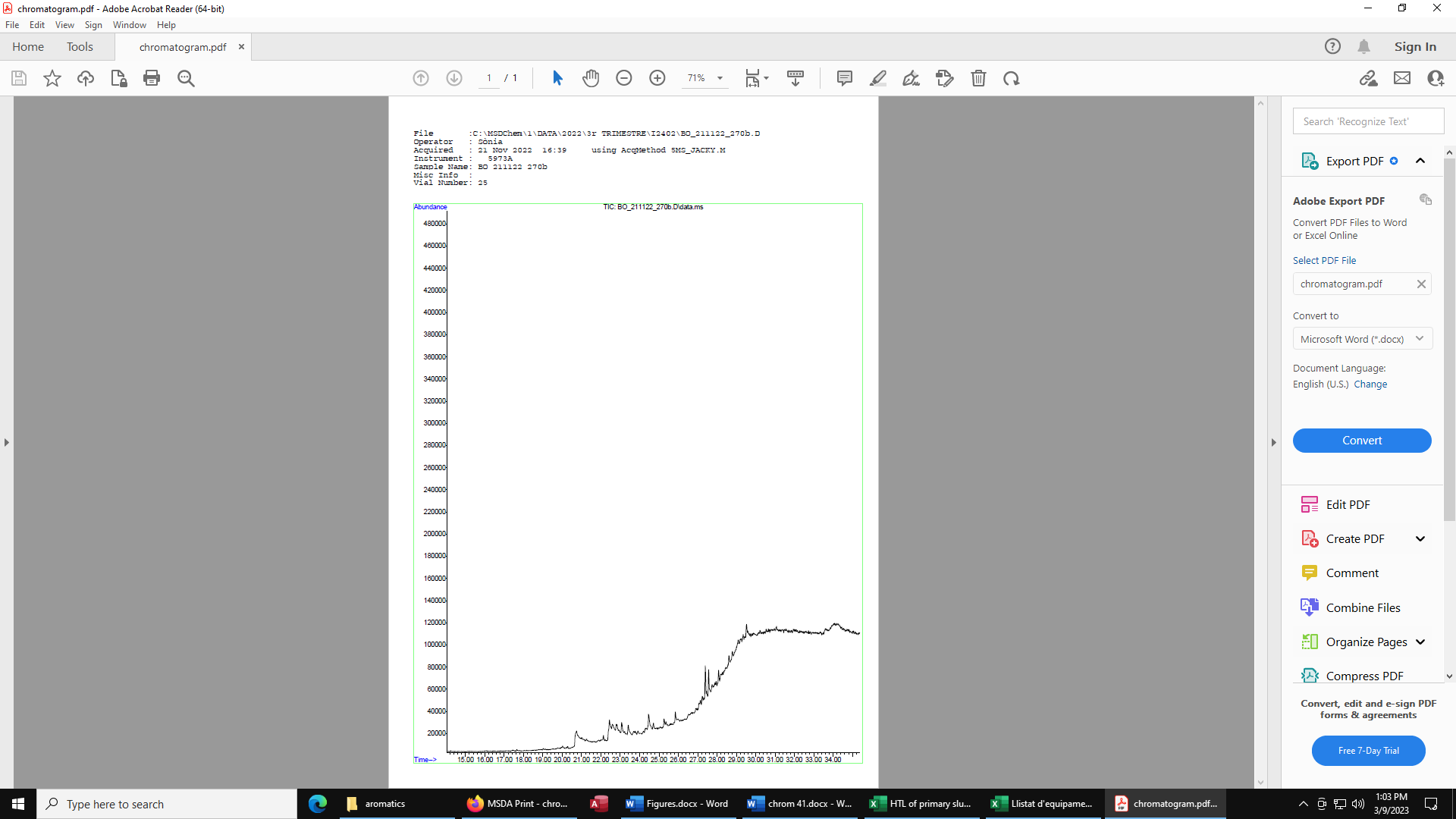 | 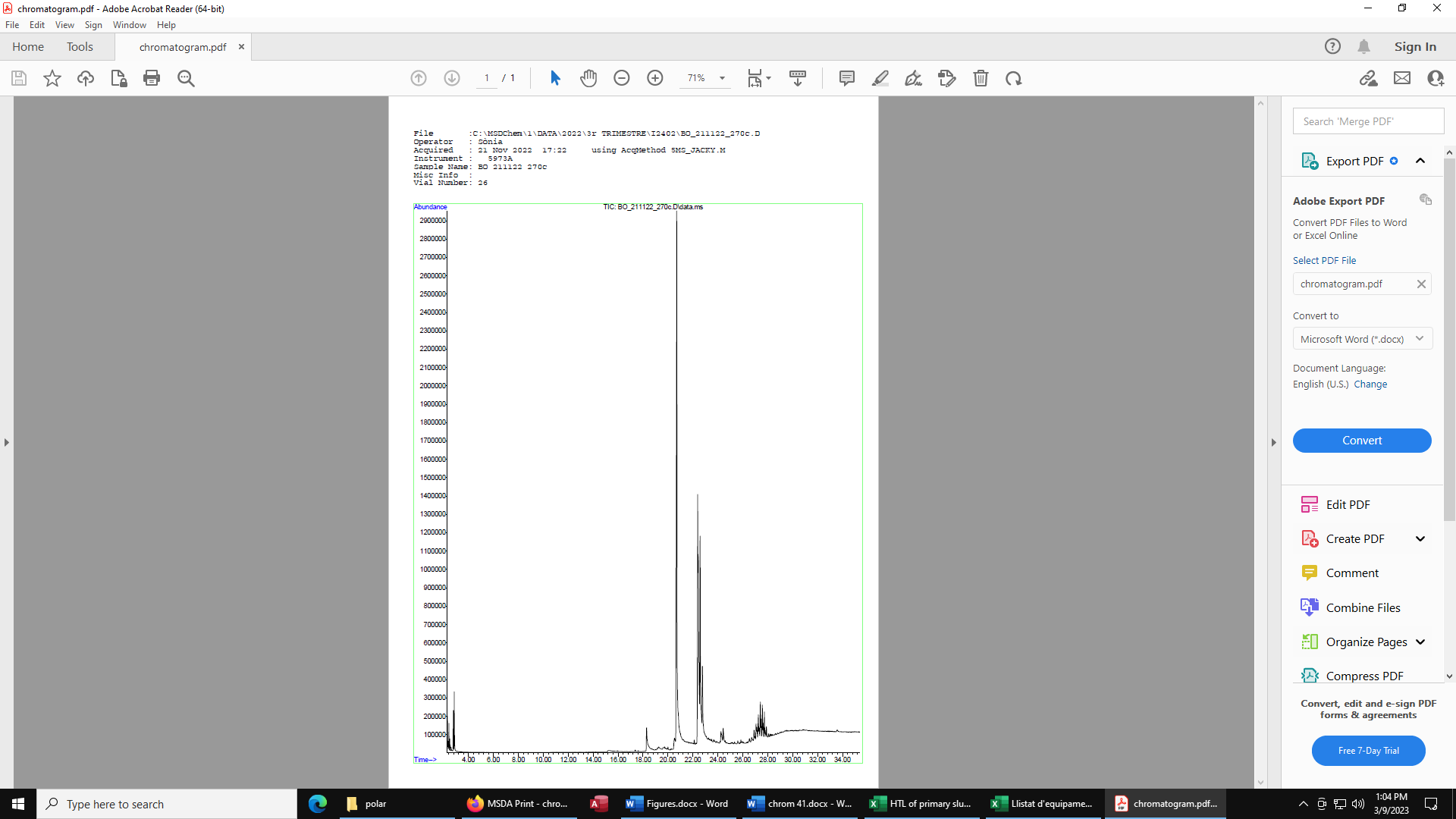 |
| (c) Aromatic oils | (d) Resins (polar oils) |

**Figure SI3.** SEM images and EDX spectra (a), (b) and (c) of ash in aqueous phase. HTL operation conditions: 300ºC of temperature, 30 min of reaction time and 100 rpm stirring rate. (a), (b) and (c) represent three different observation locations of the sample.

| 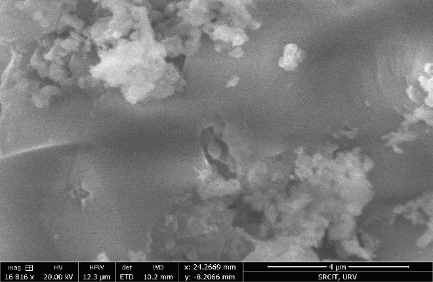  SEM image location (a) | 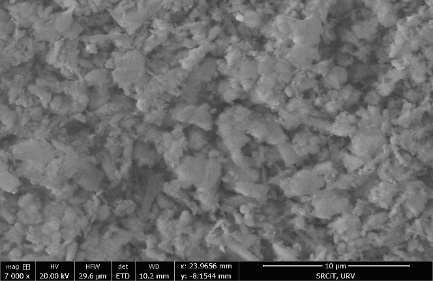  SEM image location (b) | 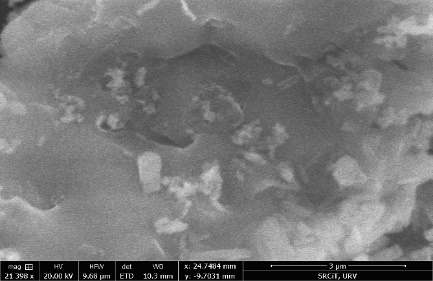  SEM image location (c) |
| --- | --- | --- |
| 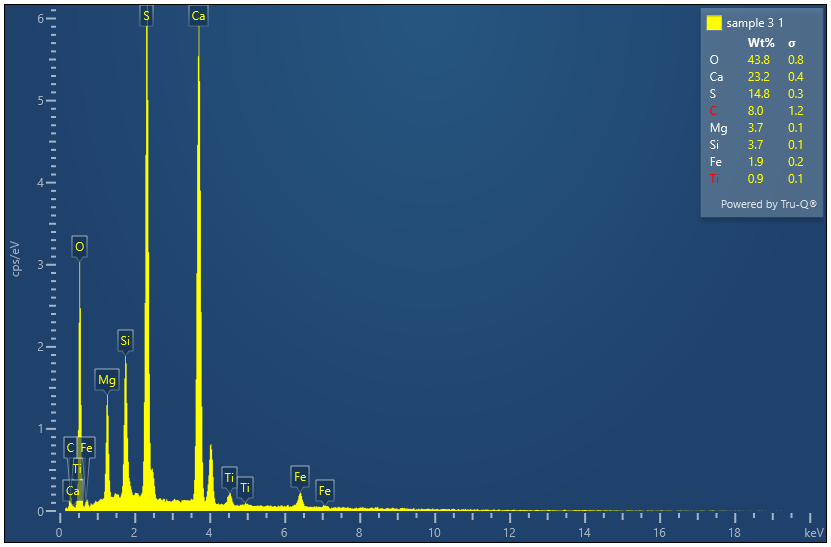  EDX spectra location (a) | 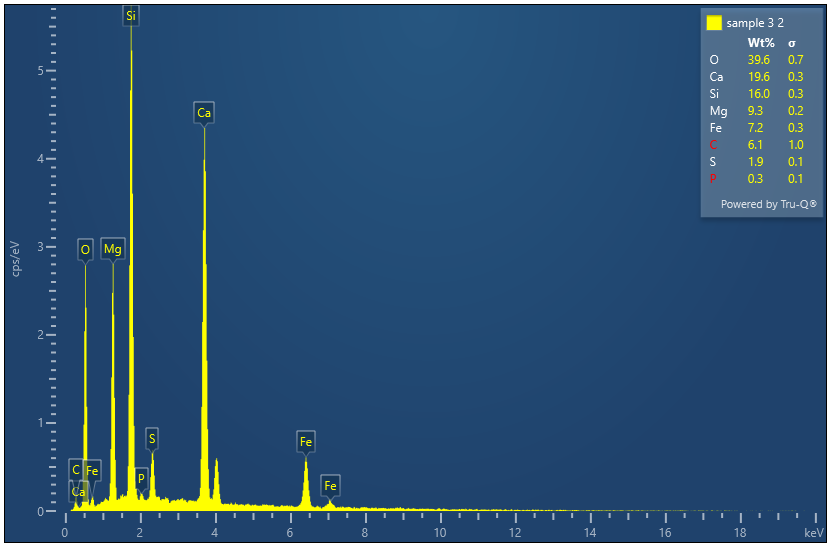  EDX spectra location (b) | 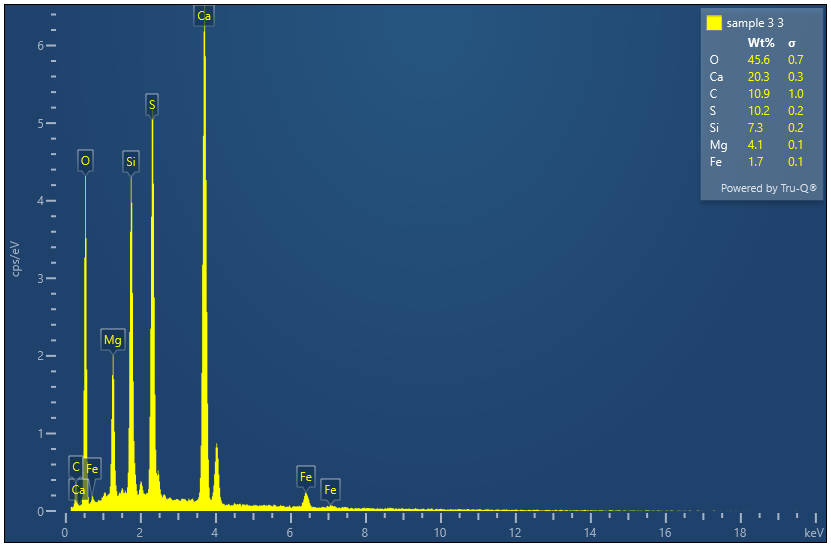  EDX spectra location (c) |

**Figure SI4.** SEM images and EDX spectra of biochar and ashes in biochar. HTL operation conditions: 300ºC of temperature, 30 min of reaction time and 100 rpm stirring rate. (a), (b), (c) and (d) represent four different observation location s of the sample.

|  | Biochar | | Ash in biochar | |
| --- | --- | --- | --- | --- |
| (a) | 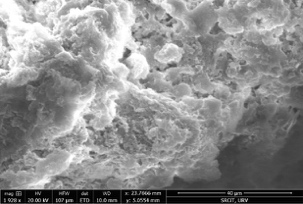 | 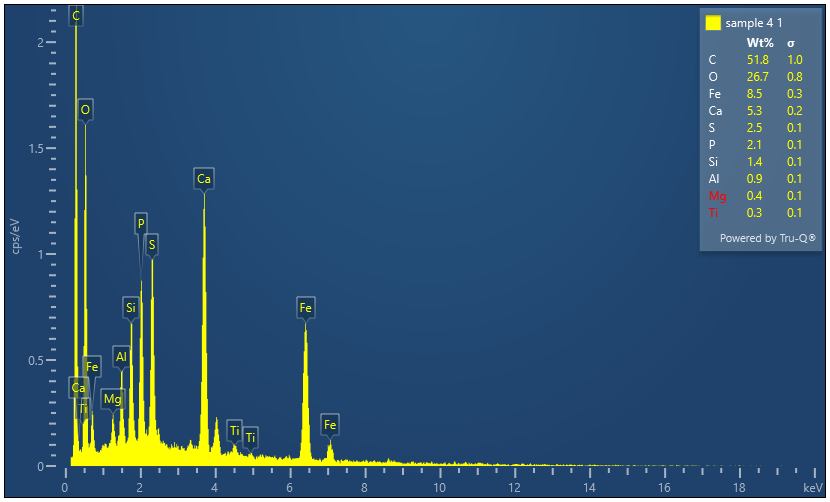 | 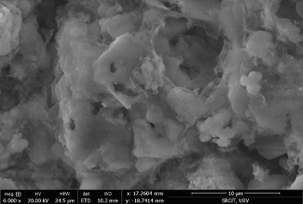 | 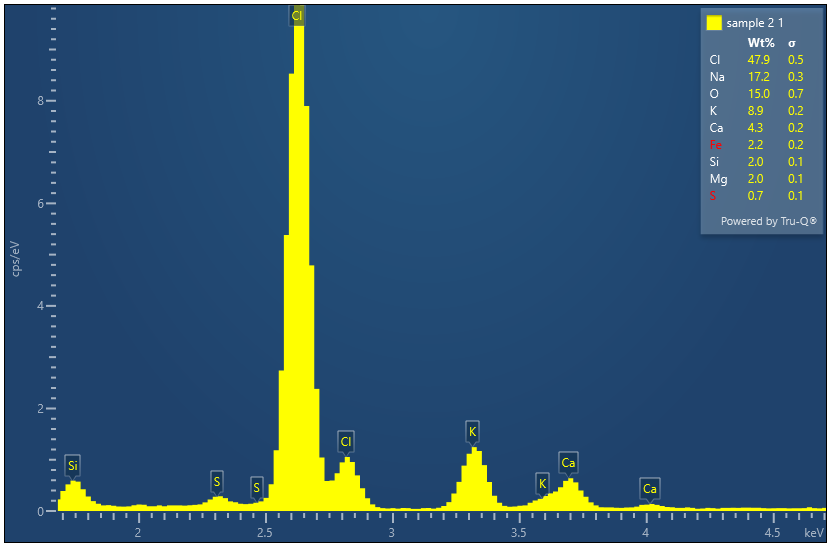 |
| (b) | 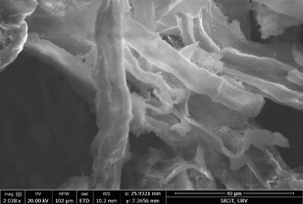 | 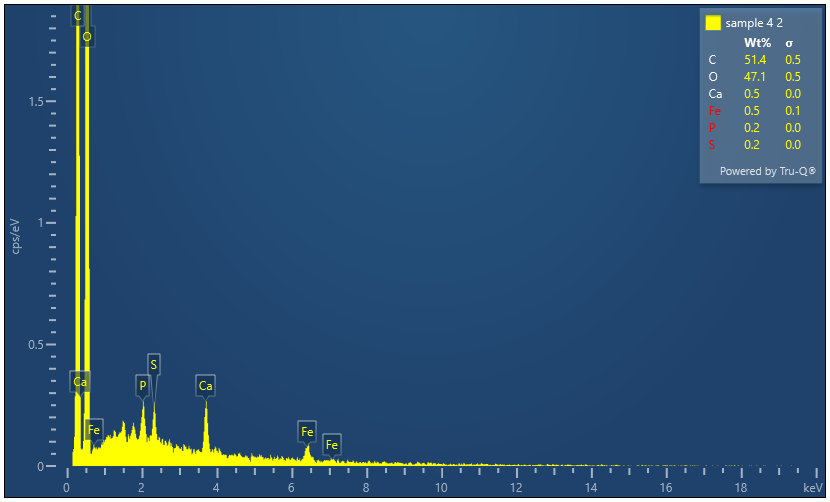 | 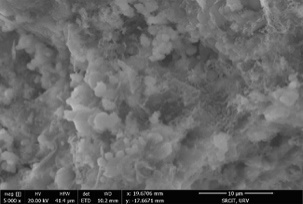 | 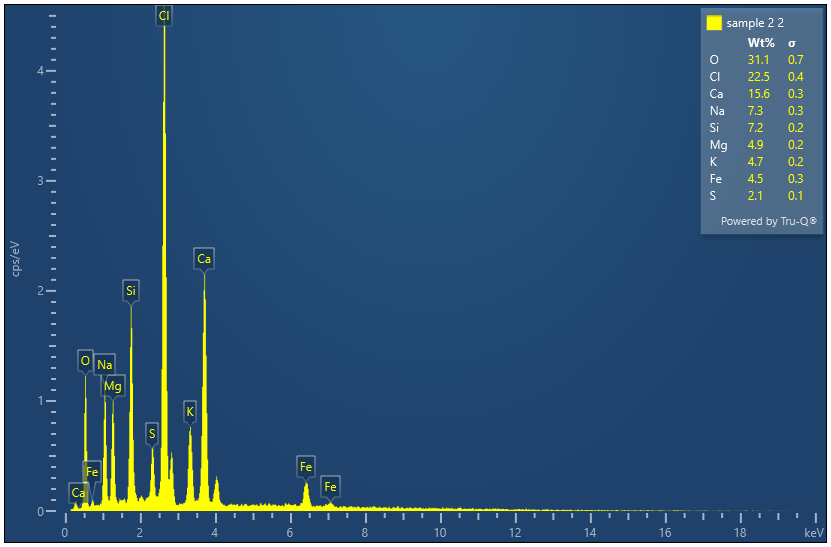 |
| (c) | 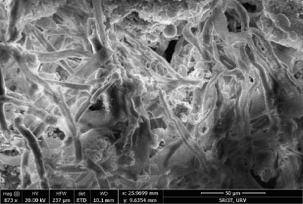 | 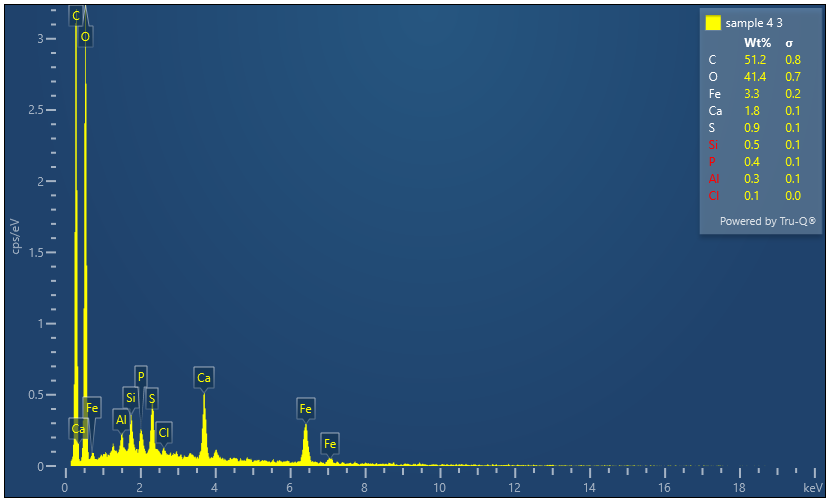 | 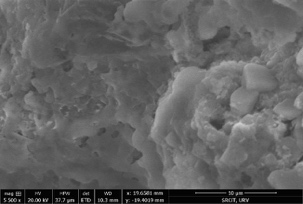 | 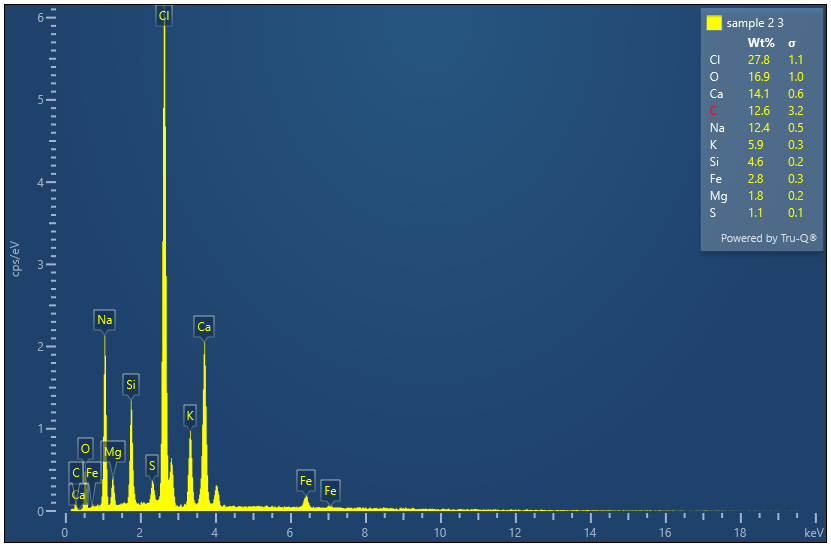 |
| (d) | 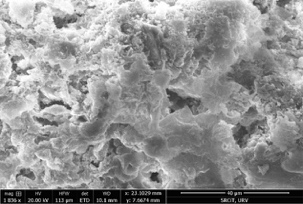 | 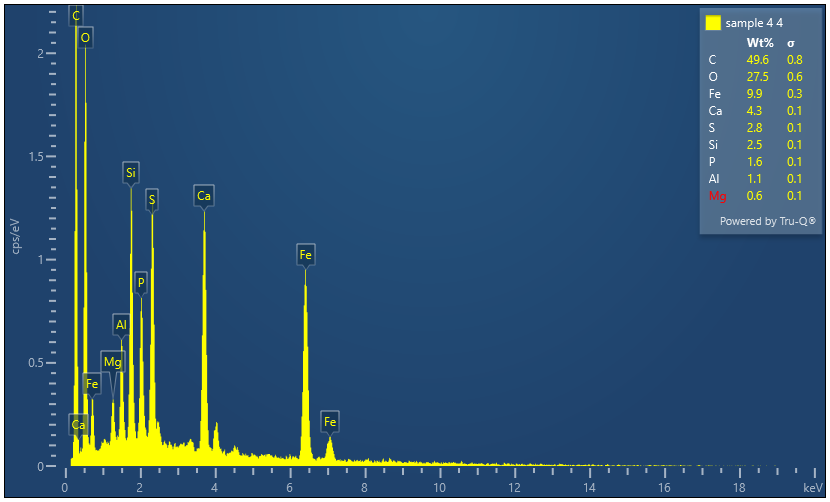 |  |  |

**Figure SI5.** Chromatogram of gaseous phase. 300ºC, 30 min of reaction time and 100 rpm stirring rate.

Column 1


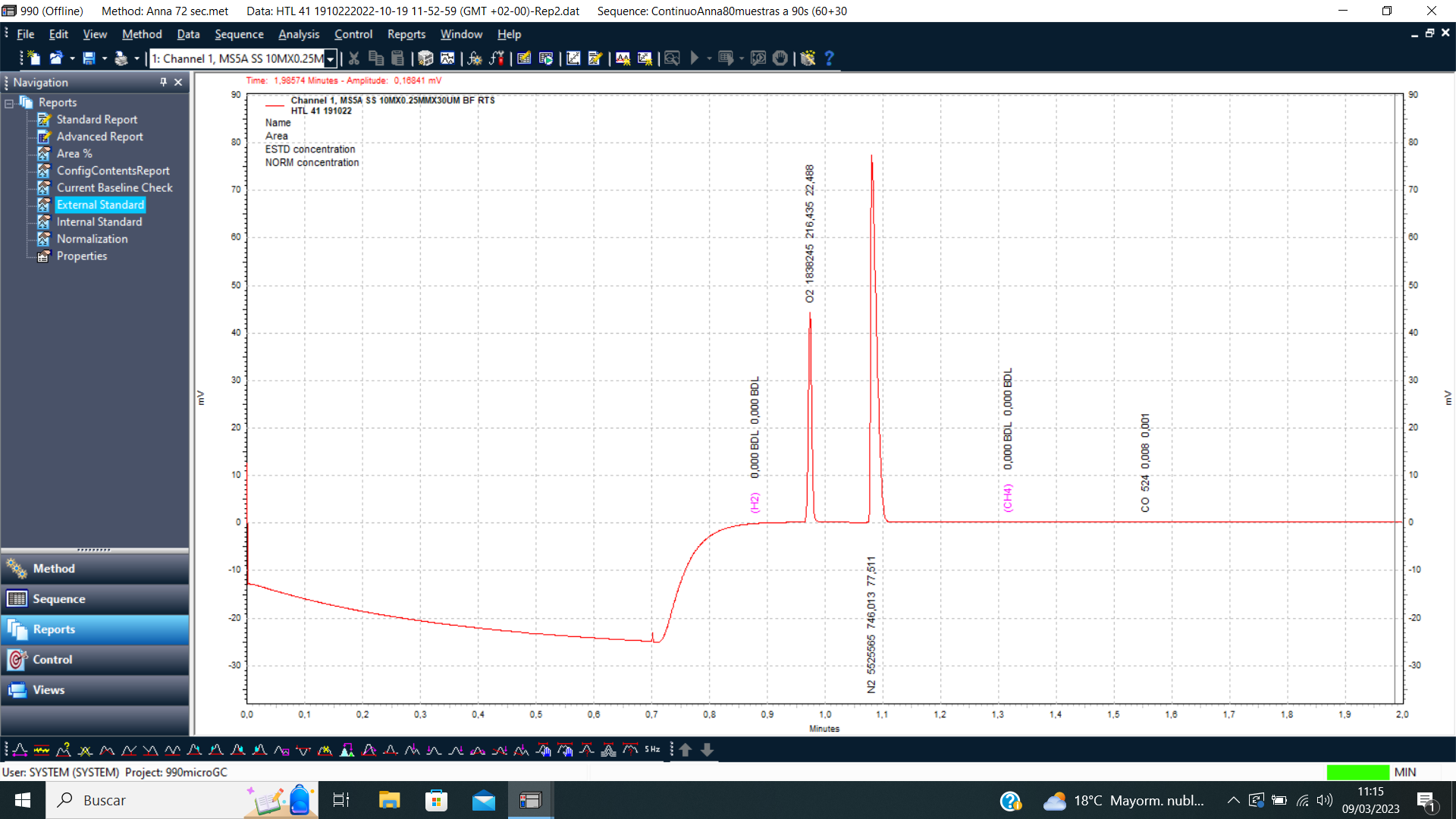


Column 2


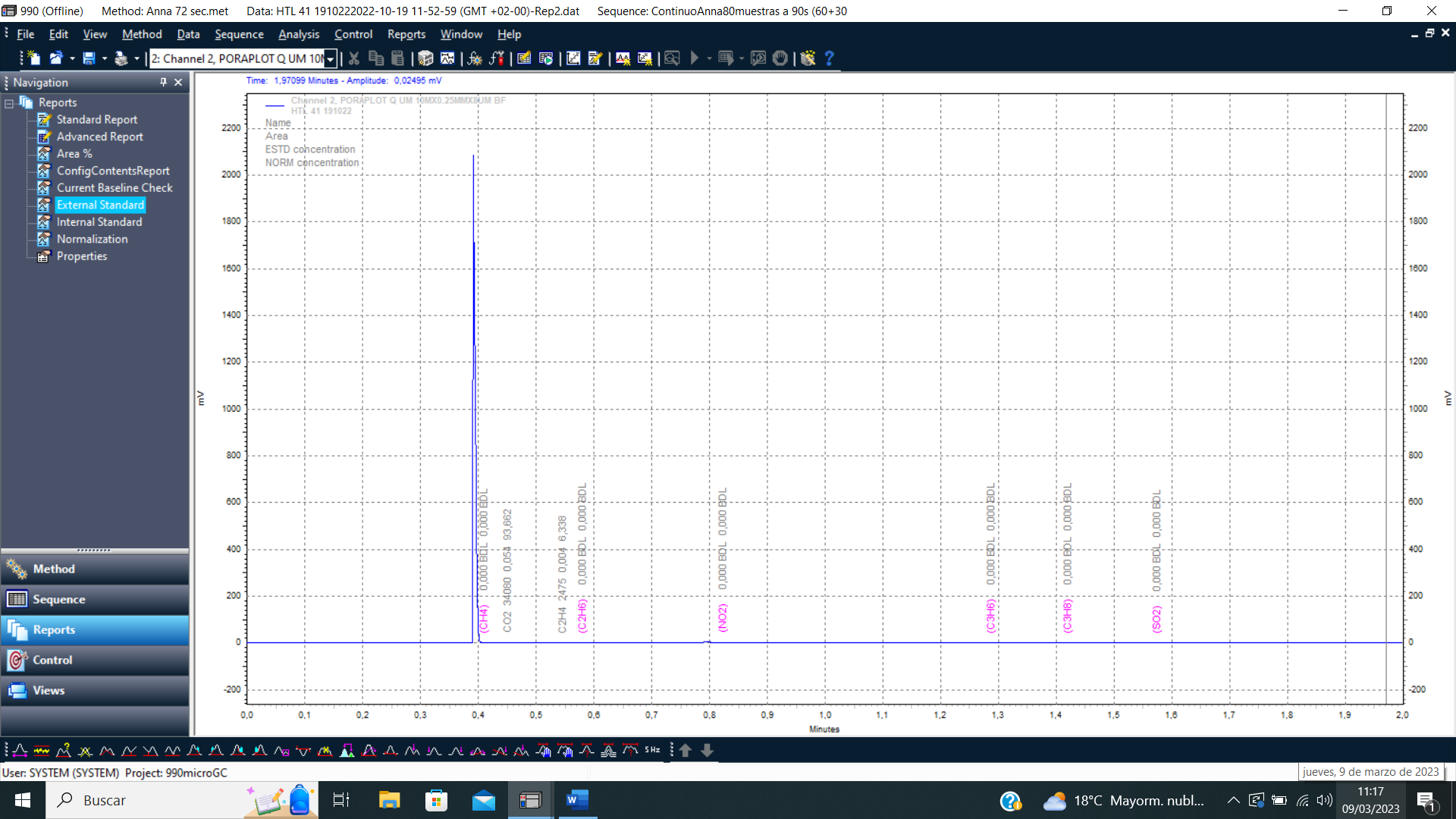

Supplement: Multimedia component 1 [file mmc1.docx]
